# Supplementary material for: The Neuromusculoskeletal Modeling Pipeline: MATLAB-based Model Personalization and Treatment Optimization Functionality for OpenSim
Source: bioRxiv. 2025 Feb 28:2024.10.30.620965. Preprint. [Version 3] doi: 10.1101/2024.10.30.620965 (PMC11601422; doi:10.1101/2024.10.30.620965)
Supplement: Supplement 1 [file media-1.zip › SupplementaryMaterial/Table S2. Constraint Terms.pdf]

Table S2. Summary of available constraint terms for Tracking, Verification, and Design Optimization.

Tracking Optimization

Constraint Term	*Component Type*
state_position_periodicity	coordinate
state_velocity_periodicity	coordinate
kinetic_consistency	load
root_segment_residual_load	load
root_segment_residual_load_periodicity	load
external_force_periodicity	force
external_moment_periodicity	moment
synergy_weight_sum	synergy_group
synergy_weight_magnitude	synergy_group

Verification Optimization

Constraint Term	*Component Type*
state_position_periodicity	coordinate
state_velocity_periodicity	coordinate
kinetic_consistency	load
root_segment_residual_load	load
root_segment_residual_load_periodicity	load
external_force_periodicity	force
external_moment_periodicity	moment

Design Optimization

Constraint Term	*Component Type*
initial_state_position	coordinate
final_state_position	coordinate
final_state_velocity	coordinate
state_position_periodicity	coordinate
state_velocity_periodicity	coordinate
kinetic_consistency	load
root_segment_residual_load	load
root_segment_residual_load_periodicity	load
external_force_periodicity	force
external_moment_periodicity	moment
limit_muscle_activation	muscle
limit_normalized_fiber_length	muscle
synergy_weight_sum	synergy_group
synergy_weight_magnitude	synergy_group
