# Supplementary material for: The Neuromusculoskeletal Modeling Pipeline: MATLAB-based Model Personalization and Treatment Optimization Functionality for OpenSim
Source: bioRxiv. 2025 Feb 28:2024.10.30.620965. Preprint. [Version 3] doi: 10.1101/2024.10.30.620965 (PMC11601422; doi:10.1101/2024.10.30.620965)
Supplement: Supplement 1 [file media-1.zip › SupplementaryMaterial/Table S1. Cost Terms.pdf]

Table S1. Summary of available cost function terms for Tracking, Verification, and Design Optimization.

Tracking Optimization

Cost Term	*Component Type*	*Max Allowable Error*	*Error Center*
generalized_coordinate_tracking	coordinate	Y	N
generalized_speed_tracking	coordinate	Y	N
joint_acceleration_minimization	coordinate	Y	N
marker_position_tracking	marker	Y	N
inverse_dynamics_load_tracking	load	Y	N
inverse_dynamics_load_minimization	load	Y	N
inverse_dynamics_slope_tracking	load	Y	N
external_force_tracking	force	Y	N
external_moment_tracking	moment	Y	N
muscle_activation_tracking	muscle	Y	N
controller_slope_minimization	controller	Y	N

Verification Optimization

Cost Term	*Component Type*	*Max Allowable Error*	*Error Center*
generalized_coordinate_tracking	coordinate	Y	N
generalized_speed_tracking	coordinate	Y	N
joint_acceleration_minimization	coordinate	Y	N
marker_position_tracking	marker	Y	N
controller_tracking	controller	Y	N
controller_slope_minimization	controller	Y	N
controller_frequency_minimization	controller	Y	N

Design Optimization

Cost Term	*Component Type*	*Max Allowable Error*	*Error Center*
generalized_coordinate_tracking	coordinate	Y	N
generalized_speed_tracking	coordinate	Y	N
joint_acceleration_minimization	coordinate	Y	N
joint_power_minimization	coordinate	Y	N
joint_energy_generation_goal	coordinate	Y	Y
joint_energy_absorption_goal	coordinate	Y	Y
marker_position_tracking	marker	Y	N
inverse_dynamics_load_tracking	load	Y	N
inverse_dynamics_slope_tracking	load	Y	N
external_force_tracking	force	Y	N
external_moment_tracking	moment	Y	N
muscle_activation_tracking	muscle	Y	N
muscle_activation_minimization	muscle	Y	N
controller_tracking	controller	Y	N
controller_slope_minimization	controller	Y	N
controller_frequency_minimization	controller	Y	N
controller_shape_tracking	controller	Y	N
angular_momentum_minimization	None	Y	N
synergy_vector_tracking	None	Y	Y
belt_speed_goal	None	Y	Y
relative_walking_speed_goal	None	Y	Y
relative_metabolic_cost_per_time	None	Y	Y
relative_metabolic_cost_per_distance	None	Y	Y
propulsive_impulse_goal	None	Y	Y
braking_impulse_goal	None	Y	Y
user_defined	Any	Y	Y
