# Supplementary material for: The Neuromusculoskeletal Modeling Pipeline: MATLAB-based Model Personalization and Treatment Optimization Functionality for OpenSim
Source: bioRxiv. 2025 Feb 28:2024.10.30.620965. Preprint. [Version 3] doi: 10.1101/2024.10.30.620965 (PMC11601422; doi:10.1101/2024.10.30.620965)
Supplement: Supplement 1 [file media-1.zip › SupplementaryMaterial/MTP/JointMomentErrors%MaxID.pdf]

Lower Body Joint Moment Matching Errors (% maximum ID moment) following Muscle-tendon Model Personalization

Joint Coordinate	Hip Flexion	Hip Adduction	Hip Rotation	Knee Angle	Ankle Angle	Subtalar Angle
Right (% max ID)	3.9	6.1	36.4	4.8	1.2	5.6
Left (% max ID)	9.1	6.1	30.3	6.6	2.3	5.7
