# Supplementary figures and images for: The Neuromusculoskeletal Modeling Pipeline: MATLAB-based Model Personalization and Treatment Optimization Functionality for OpenSim
Source: bioRxiv. 2025 Feb 28:2024.10.30.620965. Preprint. [Version 3] doi: 10.1101/2024.10.30.620965 (PMC11601422; doi:10.1101/2024.10.30.620965)

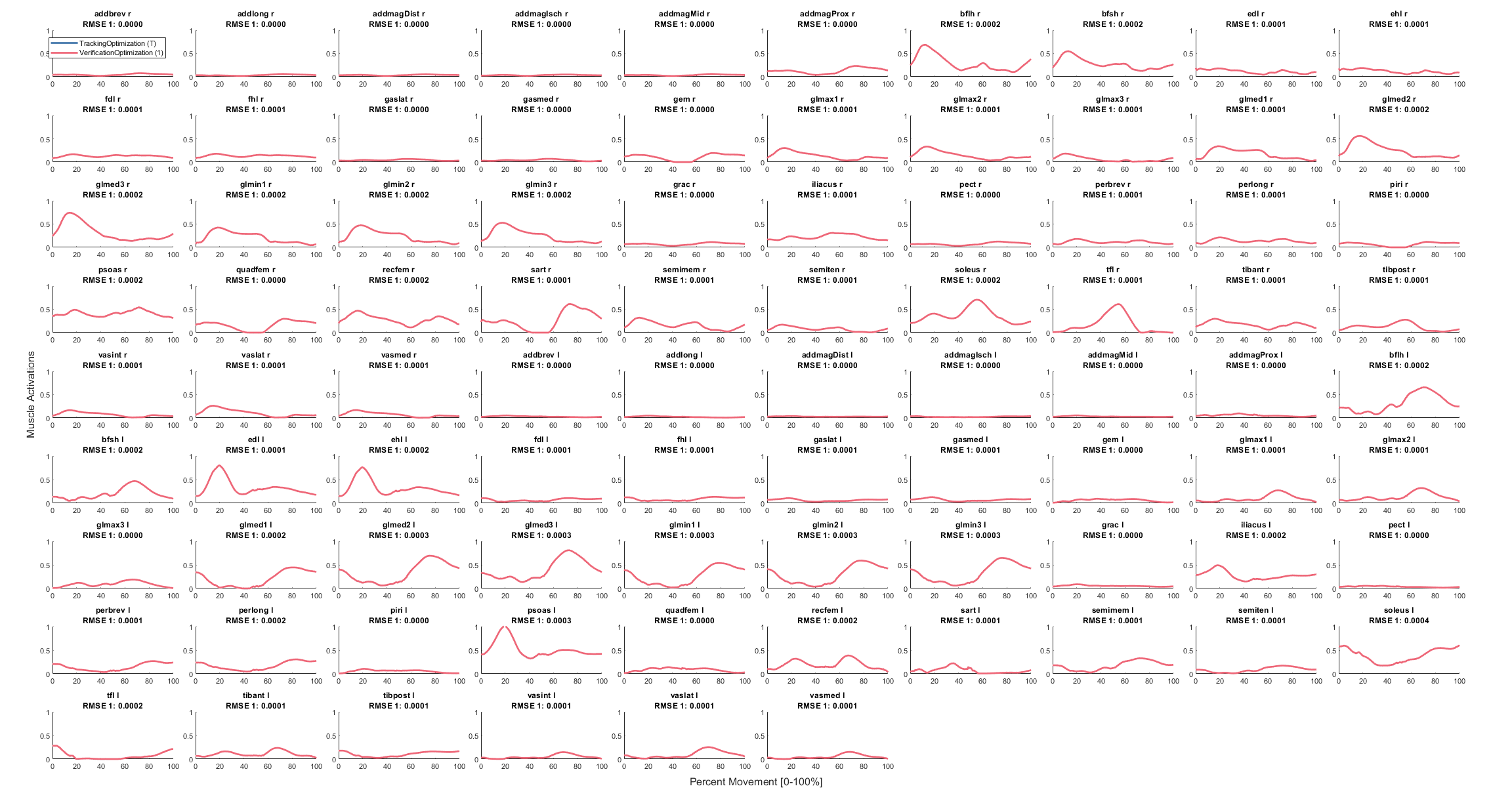

Supplement: Supplement 1 [file media-1.zip › SupplementaryMaterial/VO/activations.png]

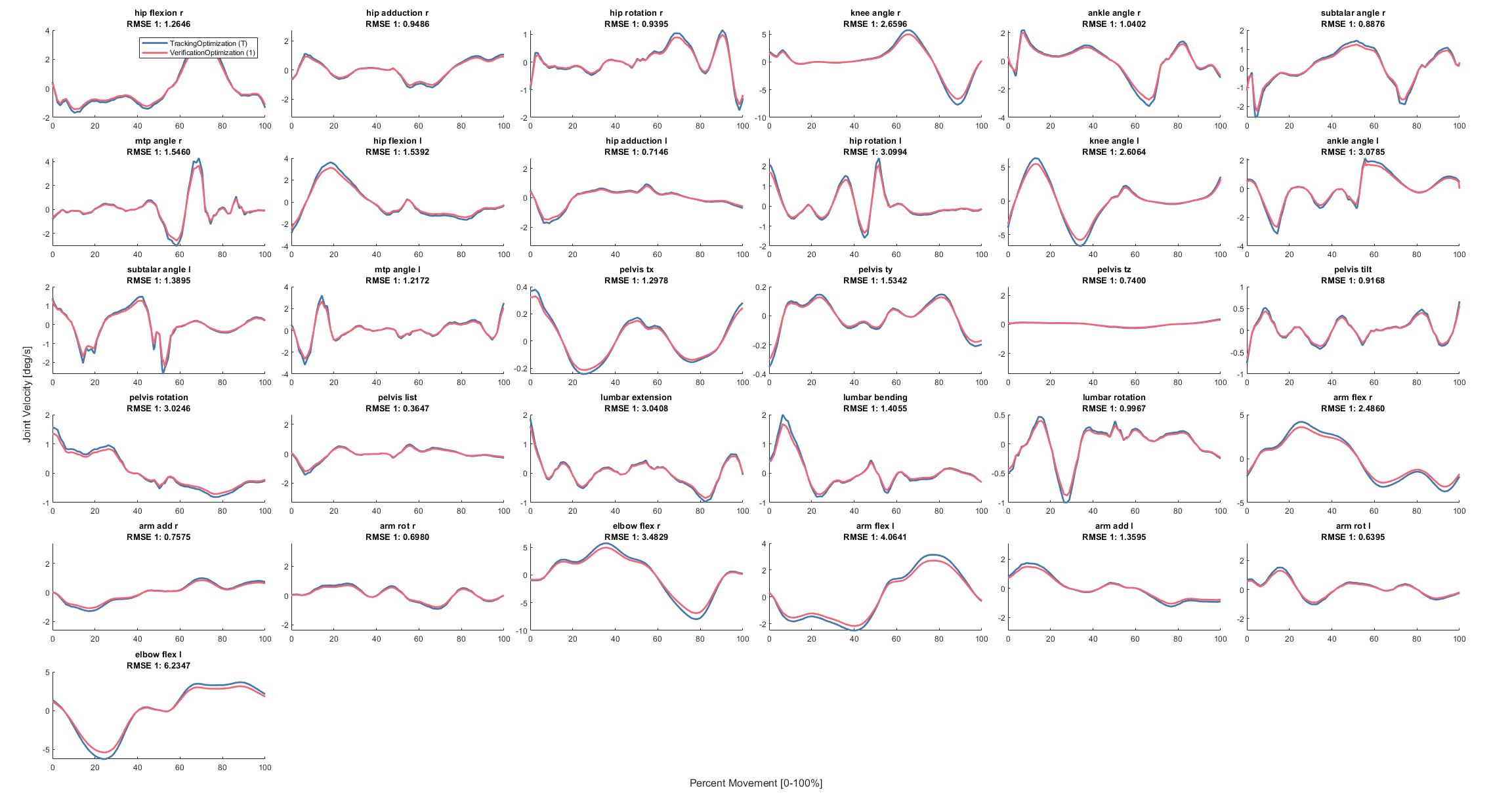

Supplement: Supplement 1 [file media-1.zip › SupplementaryMaterial/VO/jointVelocities.png]

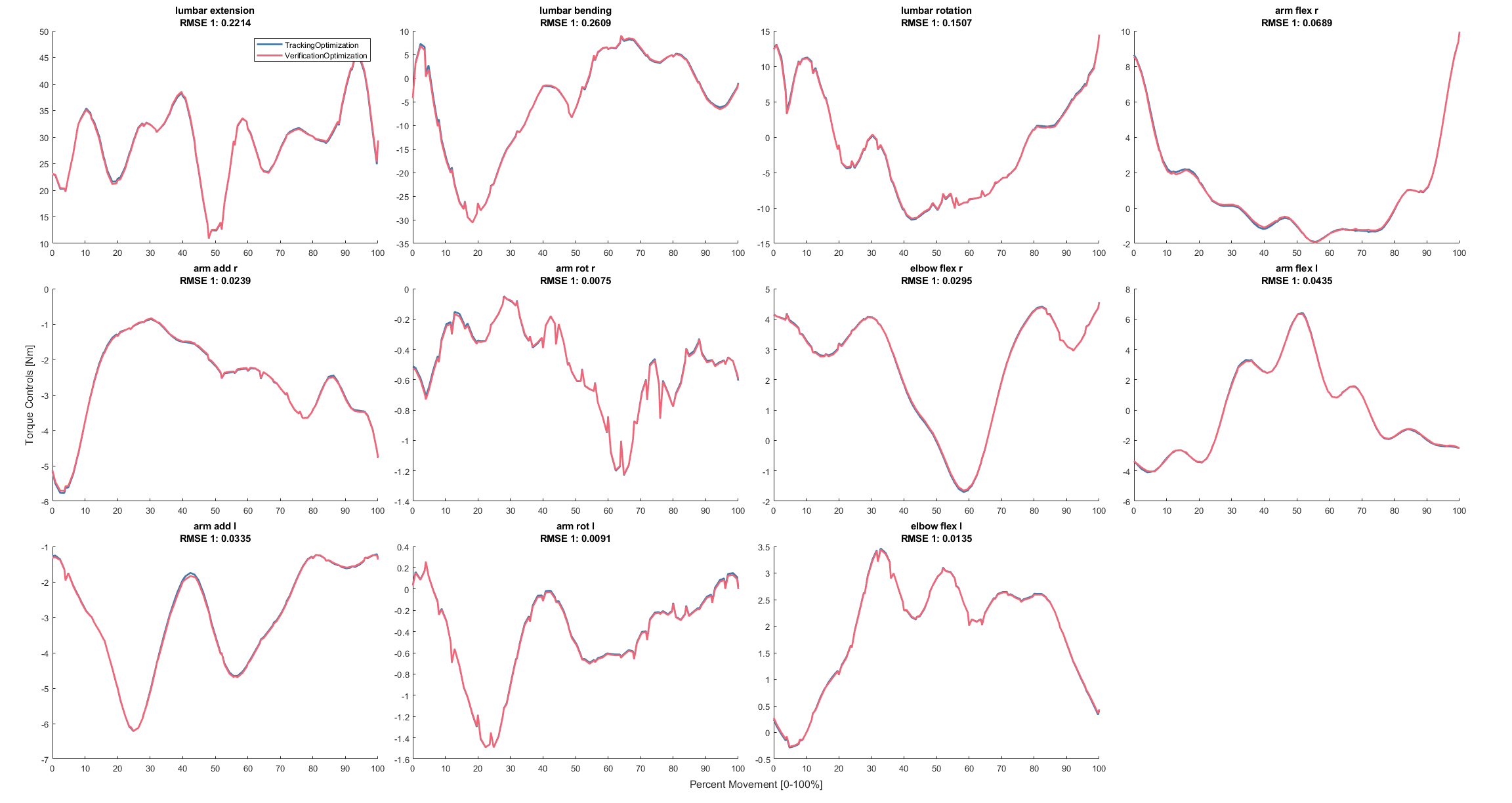

Supplement: Supplement 1 [file media-1.zip › SupplementaryMaterial/VO/torqueControls.png]

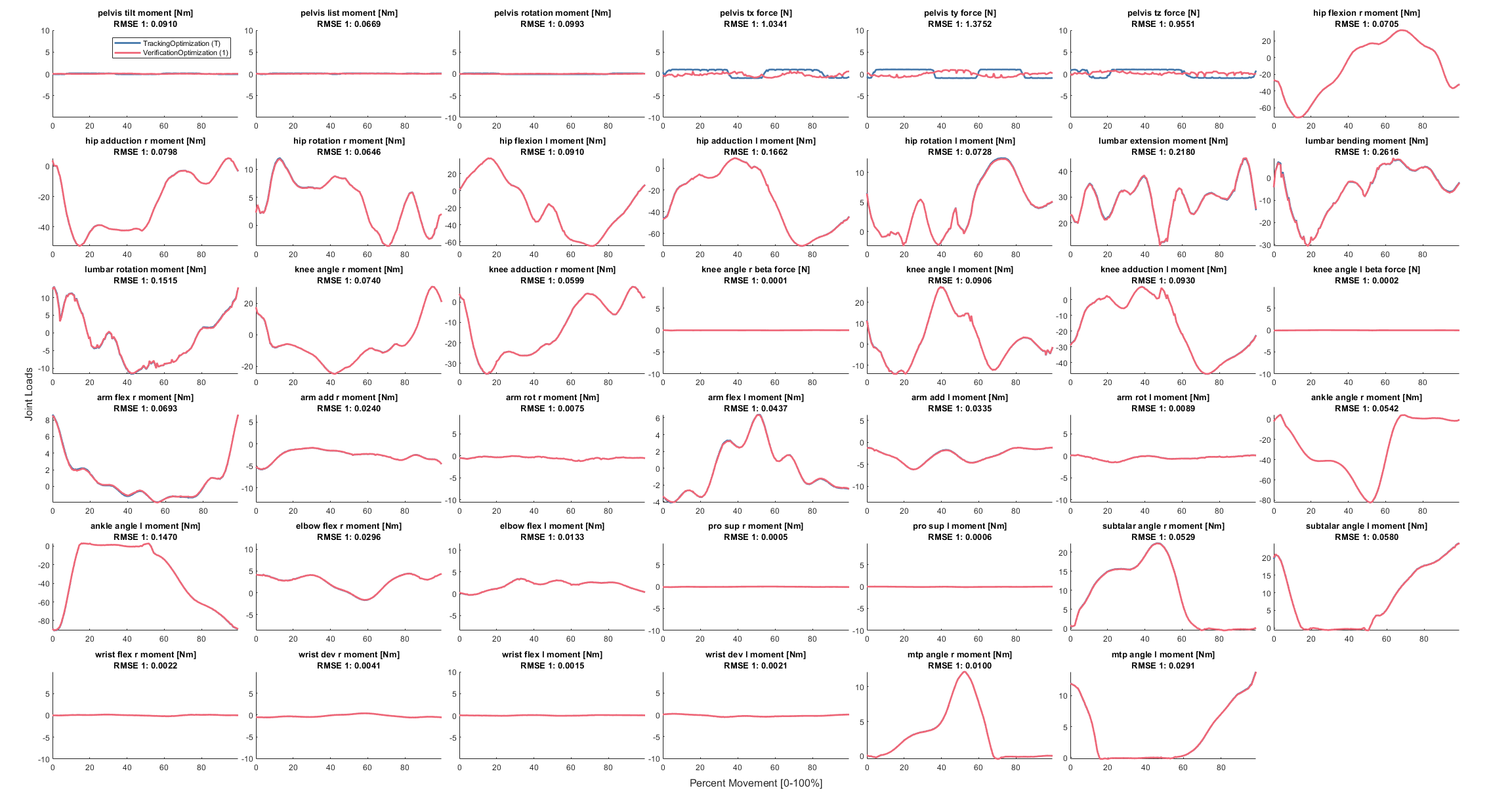

Supplement: Supplement 1 [file media-1.zip › SupplementaryMaterial/VO/jointLoads.png]

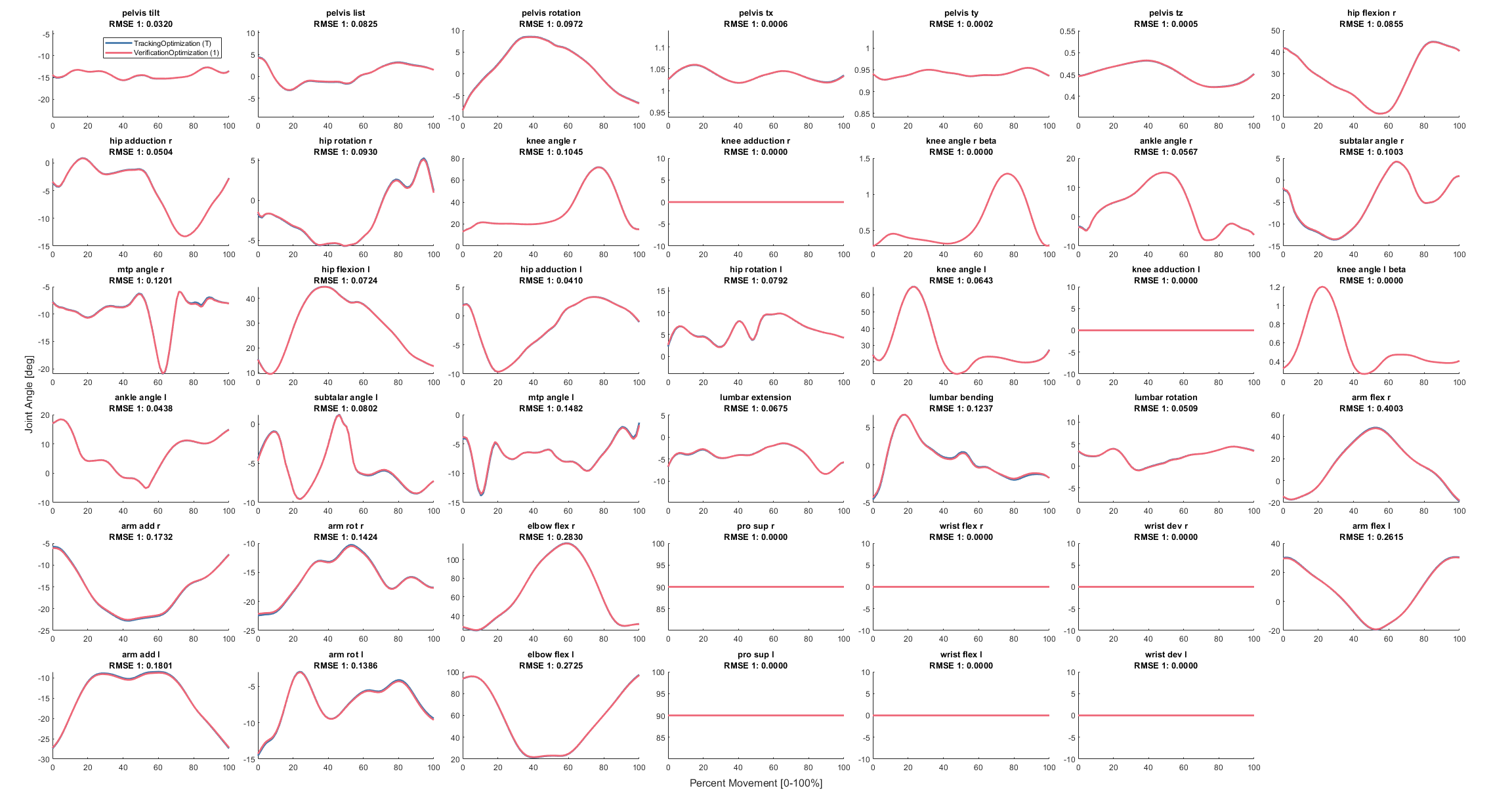

Supplement: Supplement 1 [file media-1.zip › SupplementaryMaterial/VO/jointAngles.png]

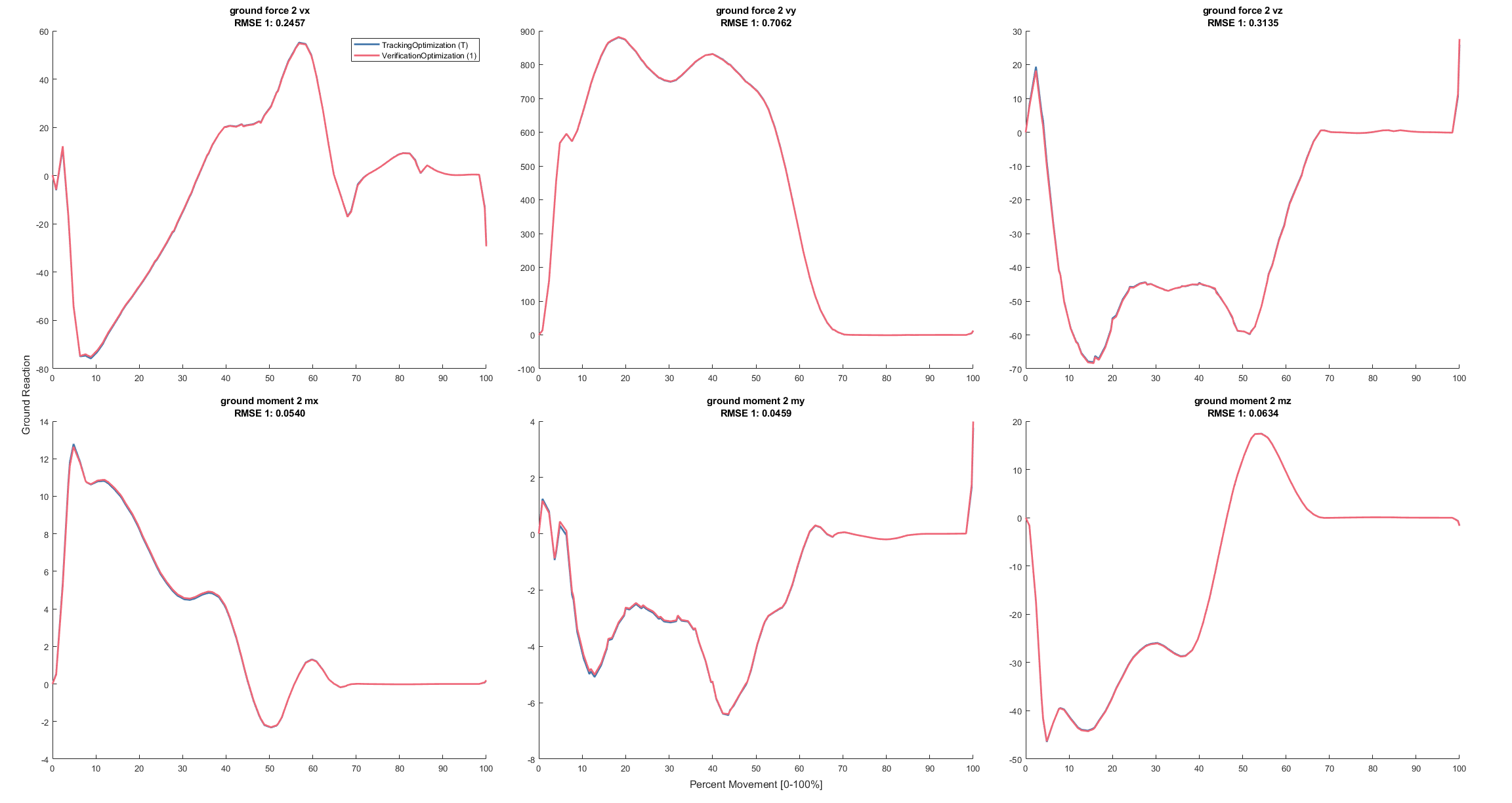

Supplement: Supplement 1 [file media-1.zip › SupplementaryMaterial/VO/foot2GroundReactions.png]

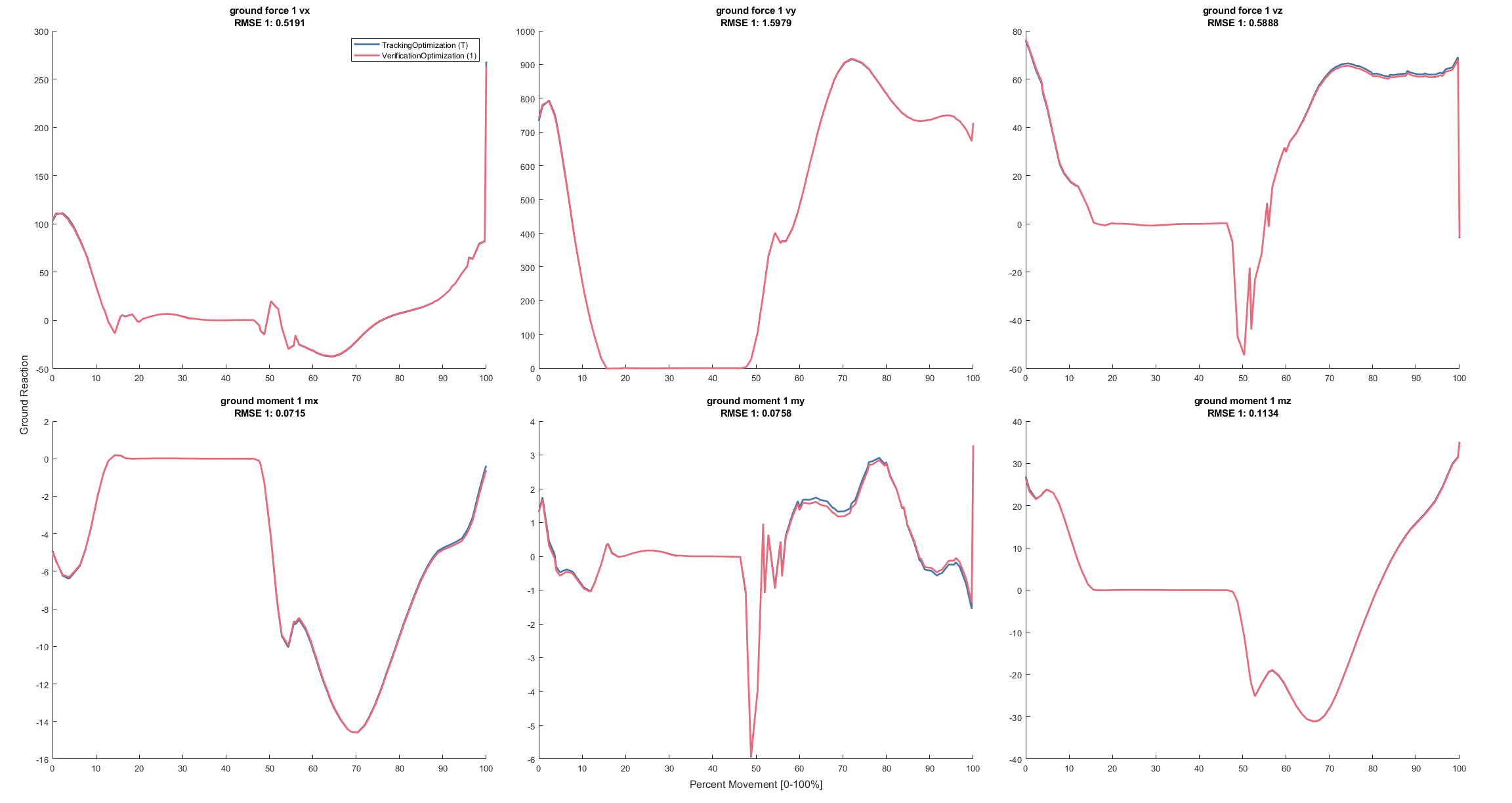

Supplement: Supplement 1 [file media-1.zip › SupplementaryMaterial/VO/foot1GroundReactions.png]

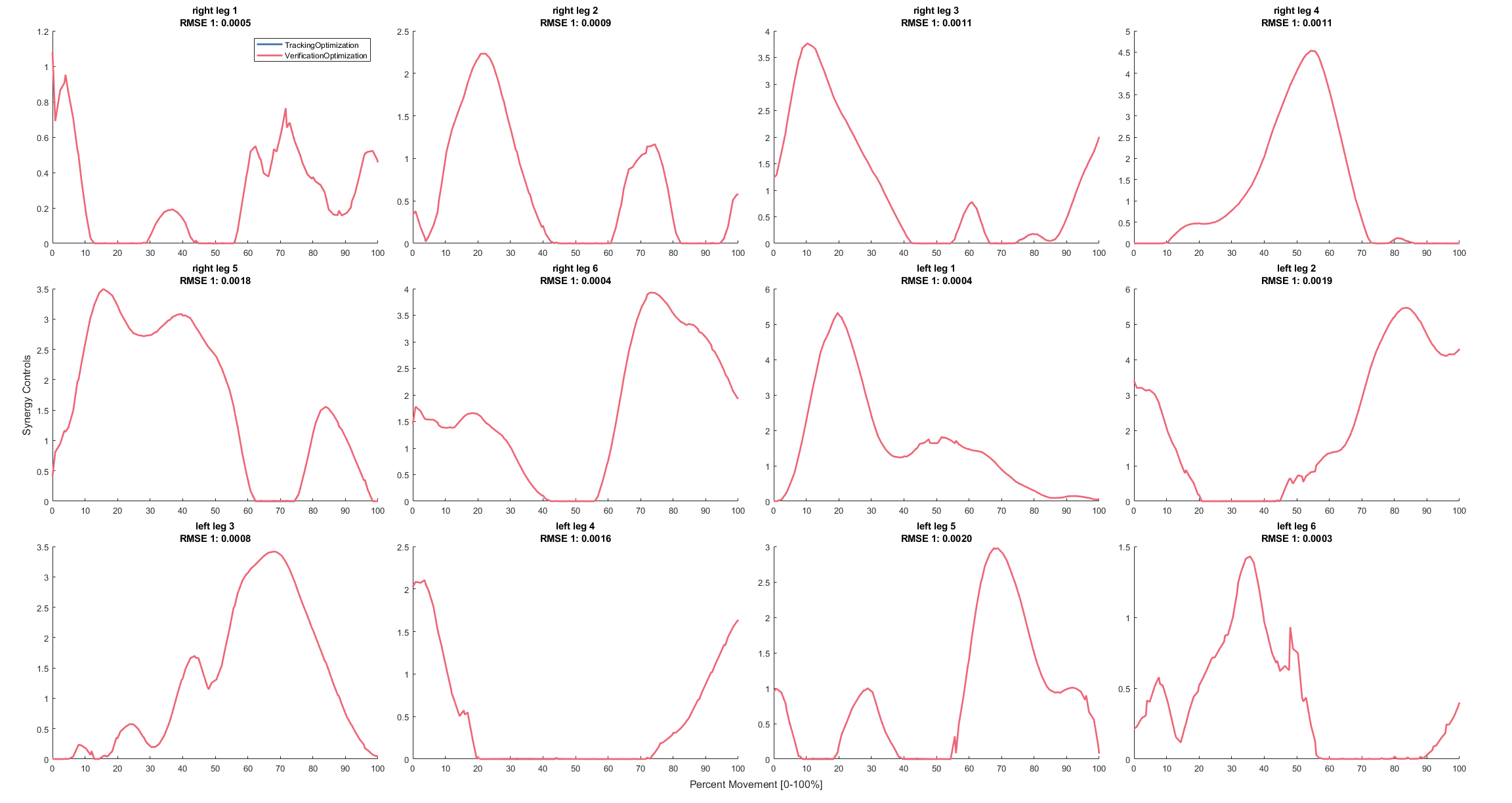

Supplement: Supplement 1 [file media-1.zip › SupplementaryMaterial/VO/synergyControls.png]

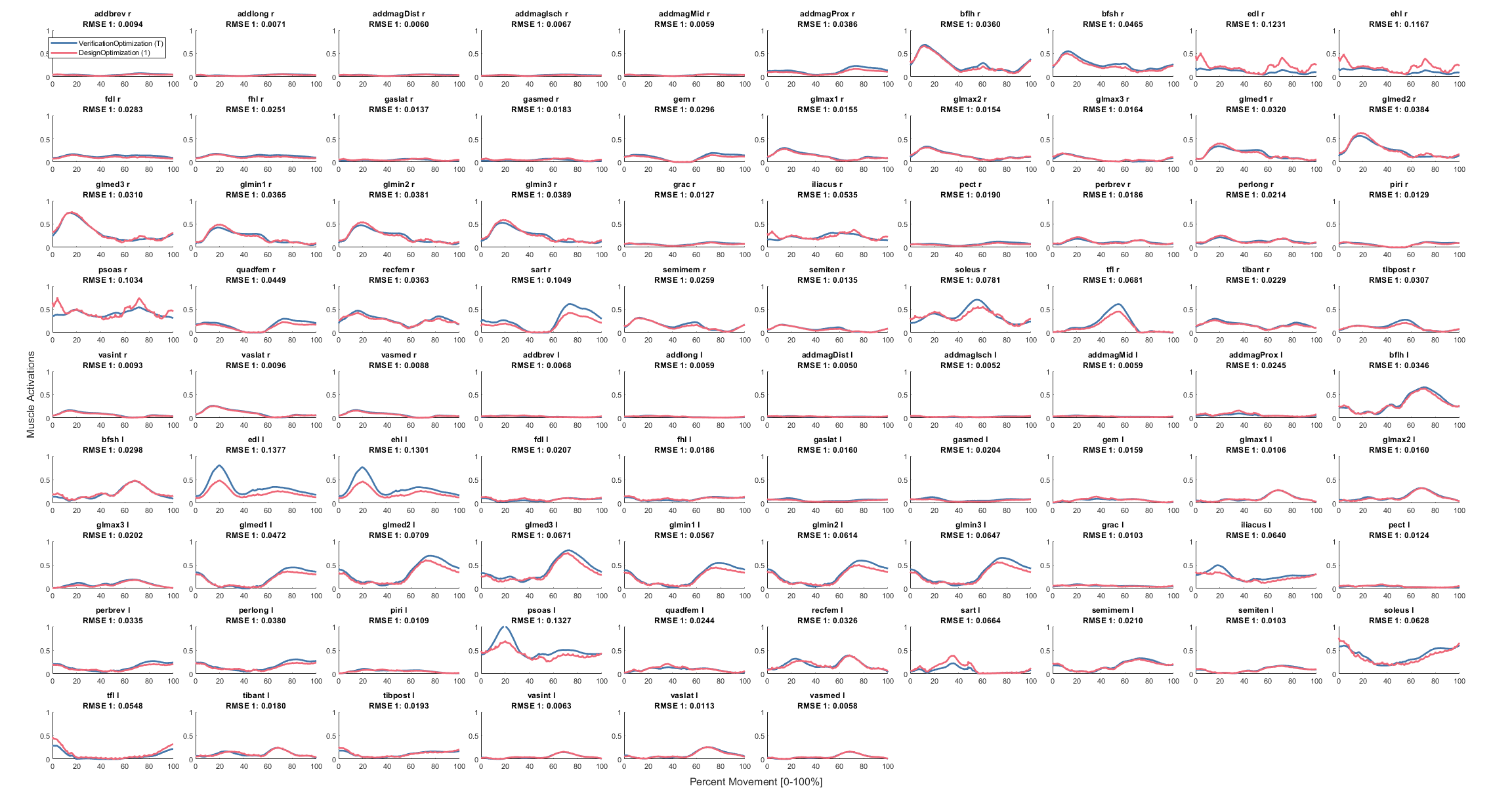

Supplement: Supplement 1 [file media-1.zip › SupplementaryMaterial/DO/muscleActivations.png]

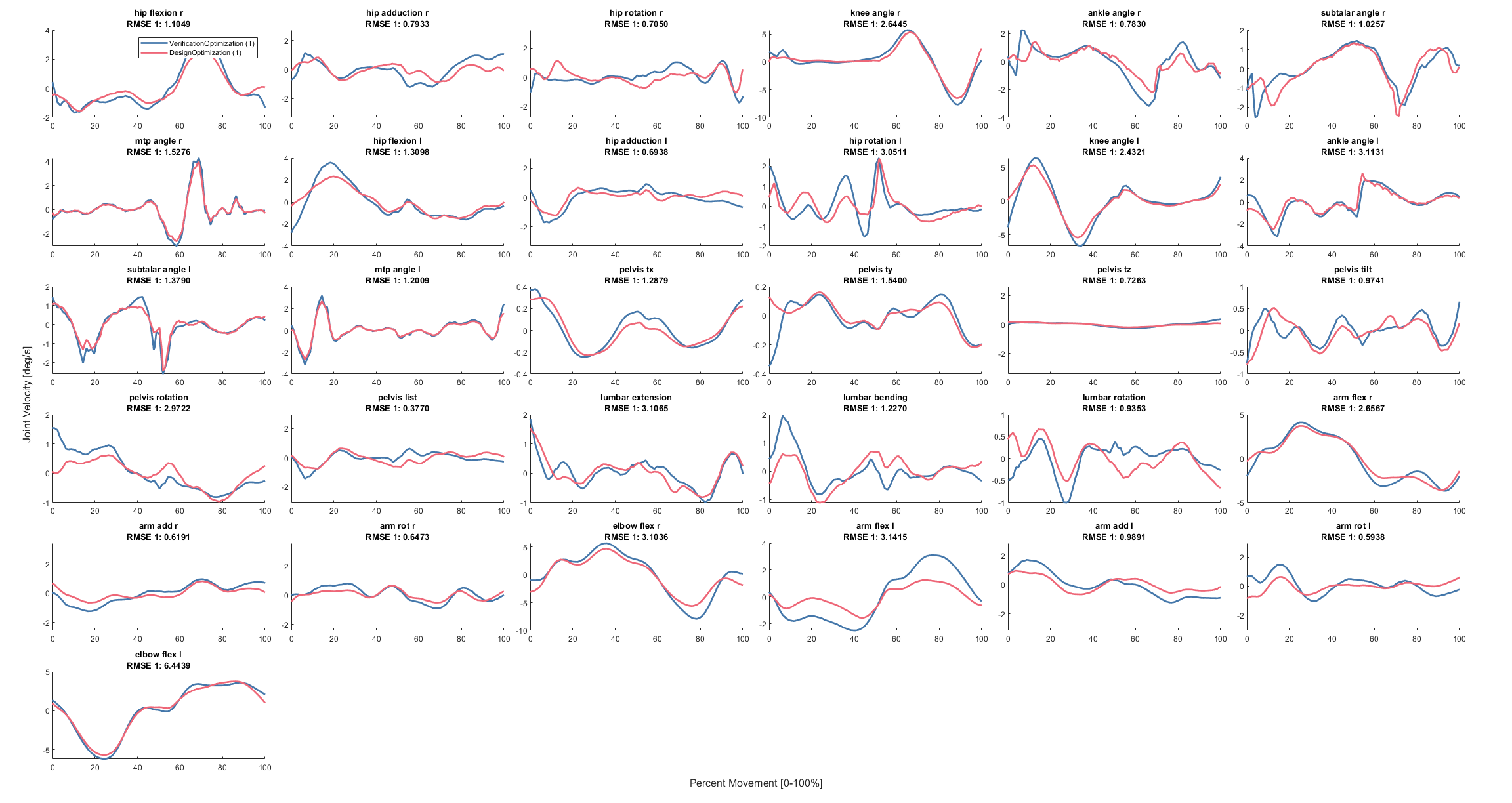

Supplement: Supplement 1 [file media-1.zip › SupplementaryMaterial/DO/jointVelocities.png]

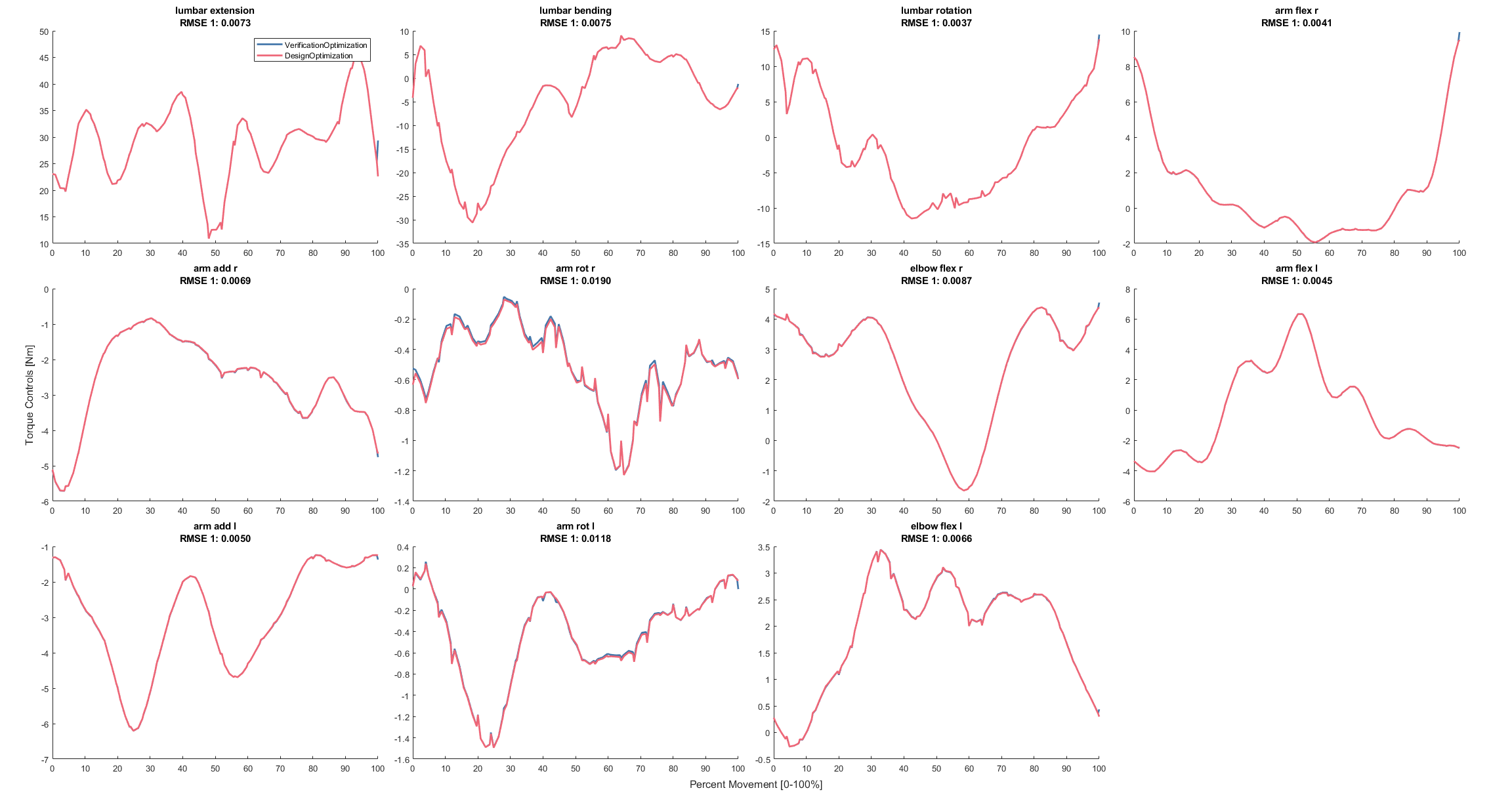

Supplement: Supplement 1 [file media-1.zip › SupplementaryMaterial/DO/torqueControls.png]

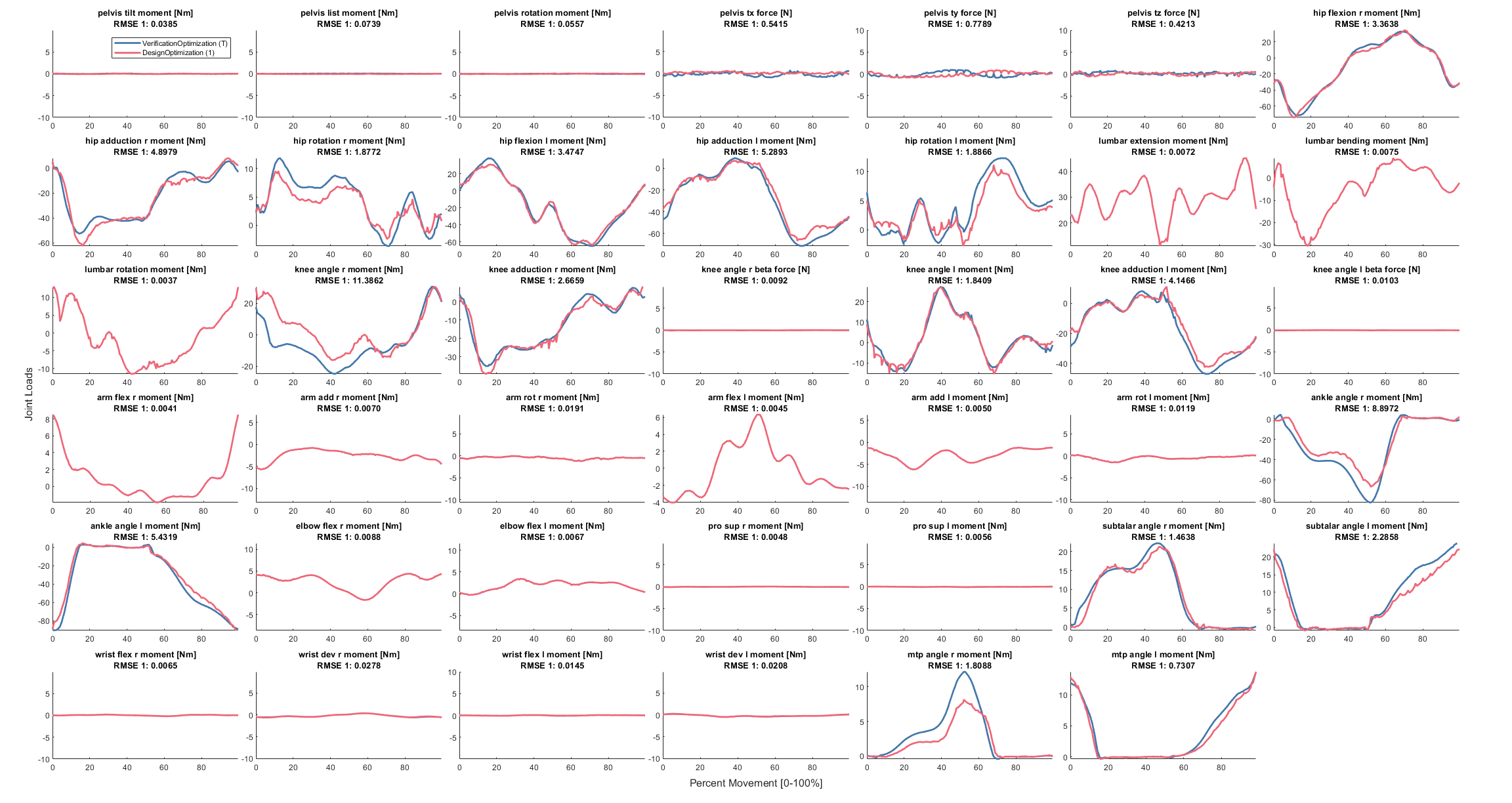

Supplement: Supplement 1 [file media-1.zip › SupplementaryMaterial/DO/jointLoads.png]

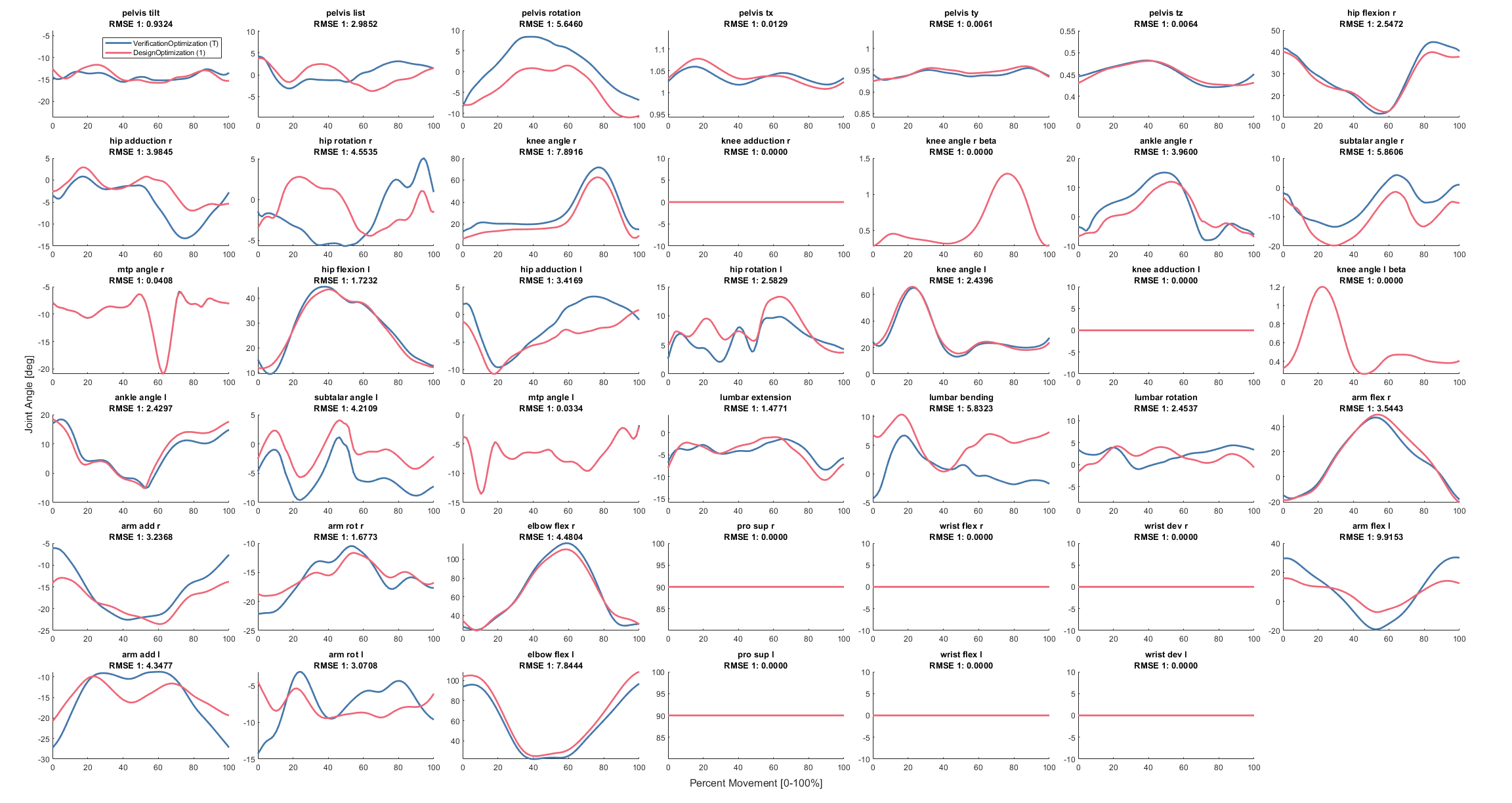

Supplement: Supplement 1 [file media-1.zip › SupplementaryMaterial/DO/jointAngles.png]

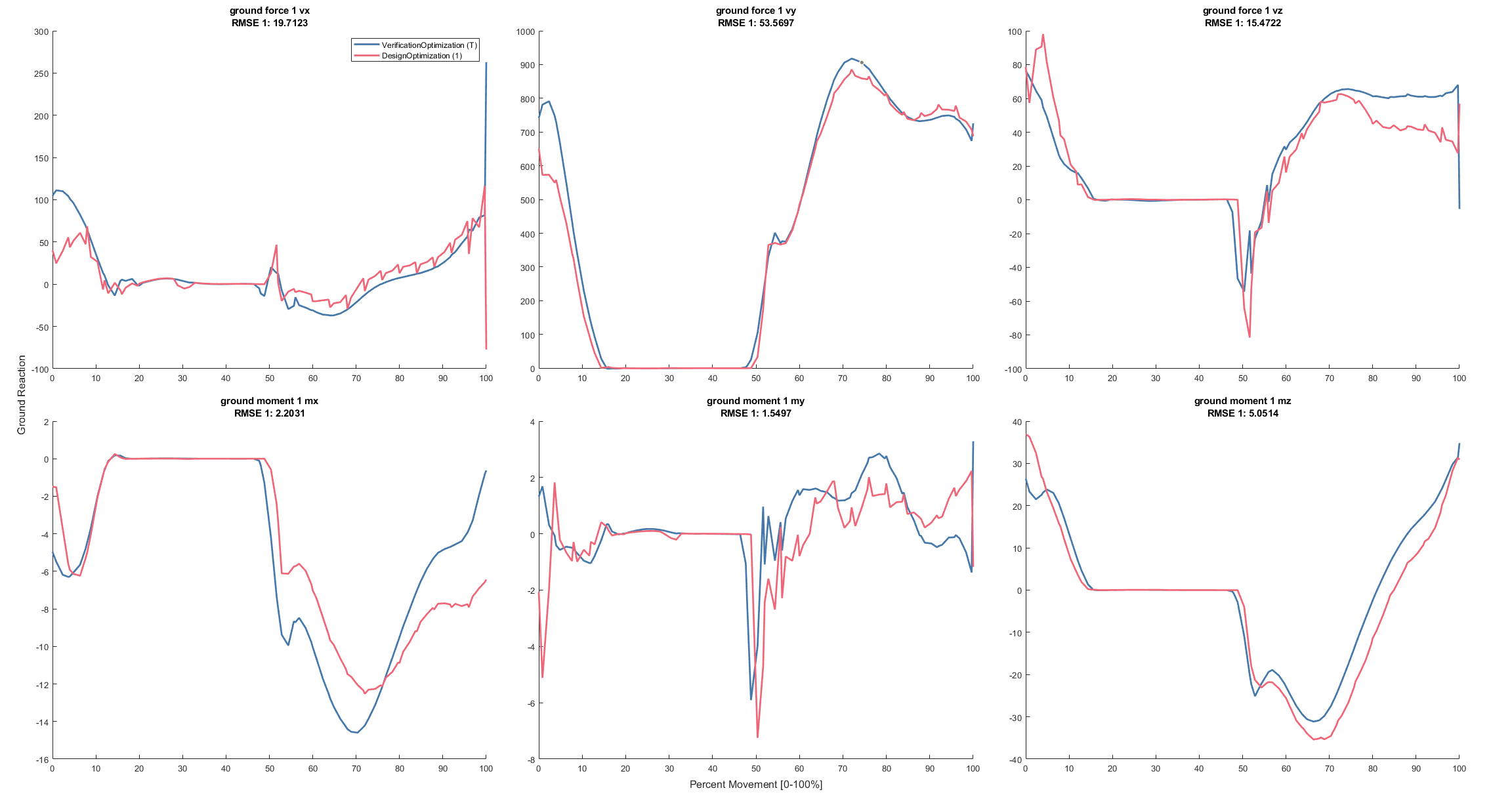

Supplement: Supplement 1 [file media-1.zip › SupplementaryMaterial/DO/foot1GroundReactions.png]

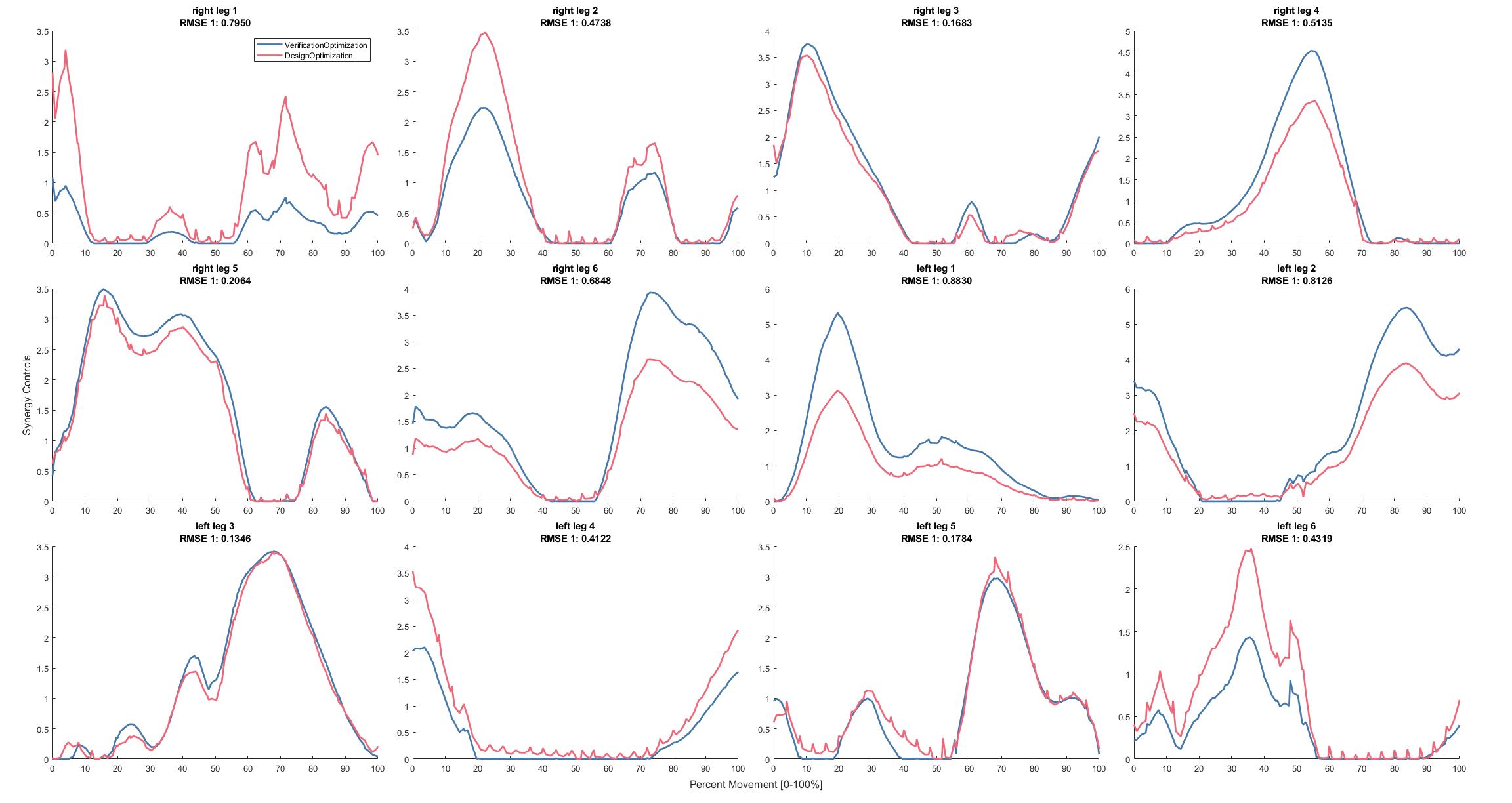

Supplement: Supplement 1 [file media-1.zip › SupplementaryMaterial/DO/synergyControls.png]

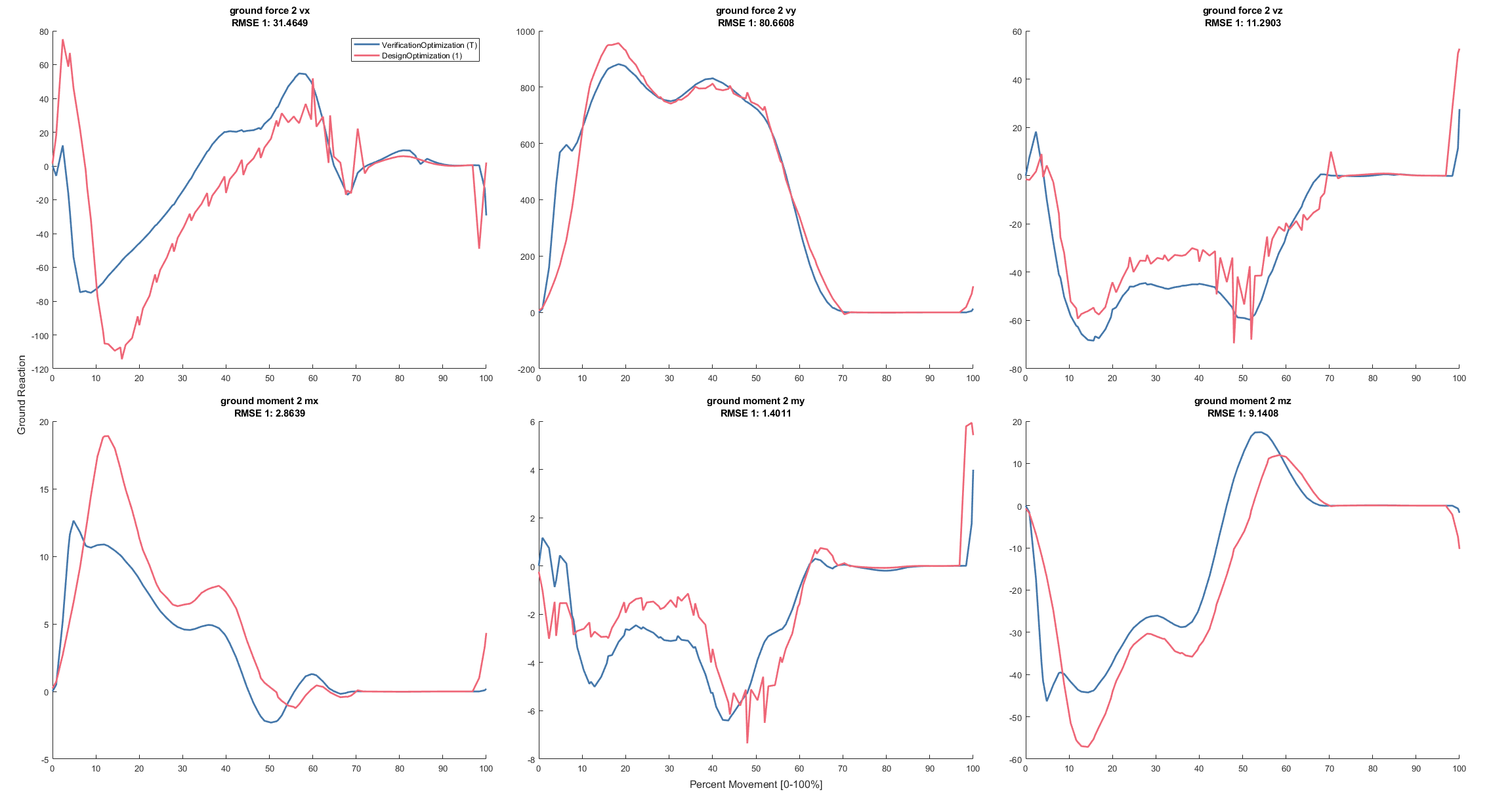

Supplement: Supplement 1 [file media-1.zip › SupplementaryMaterial/DO/foot2GroundReactionsa.png]

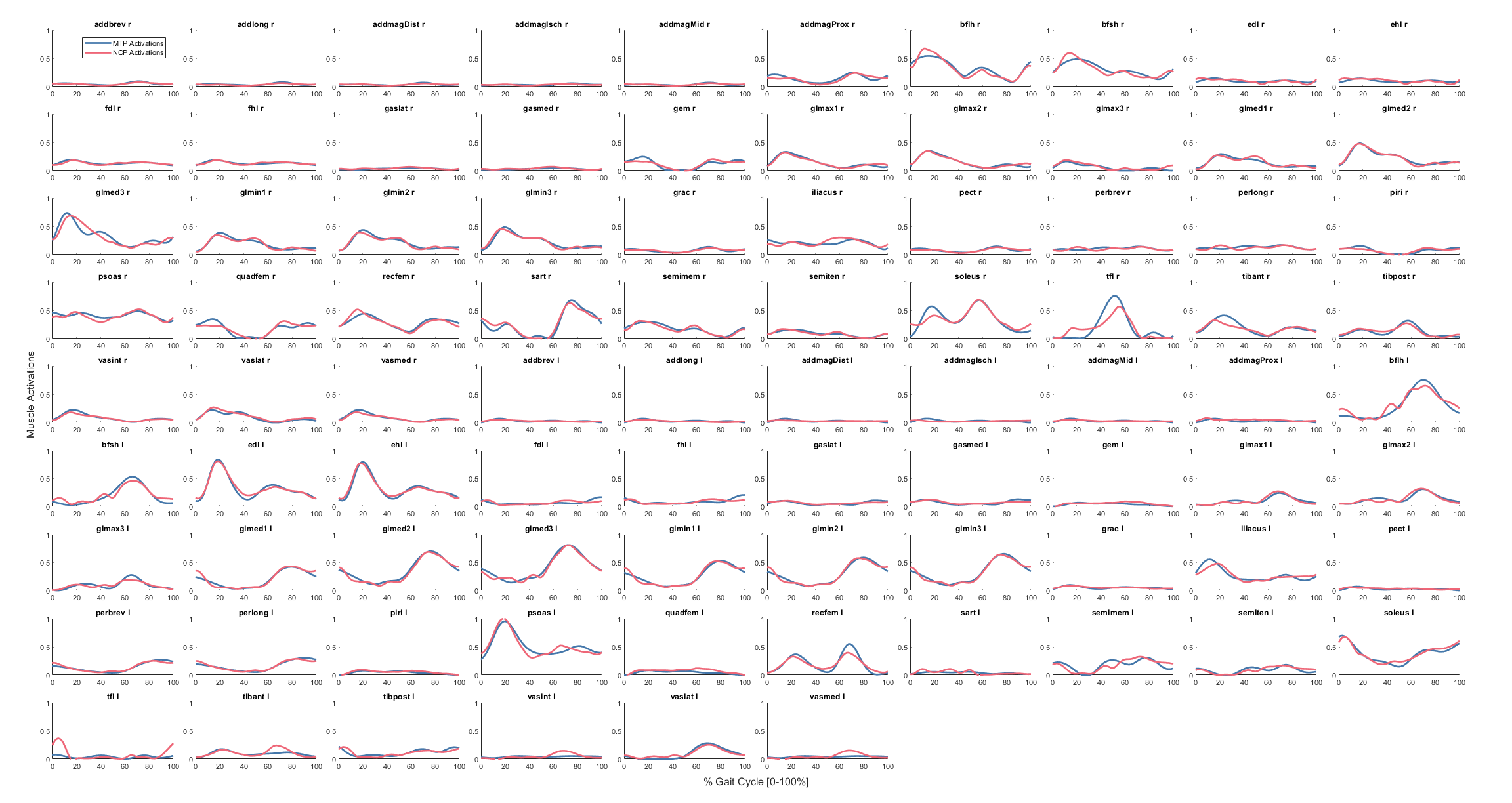

Supplement: Supplement 1 [file media-1.zip › SupplementaryMaterial/NCP/activations.png]

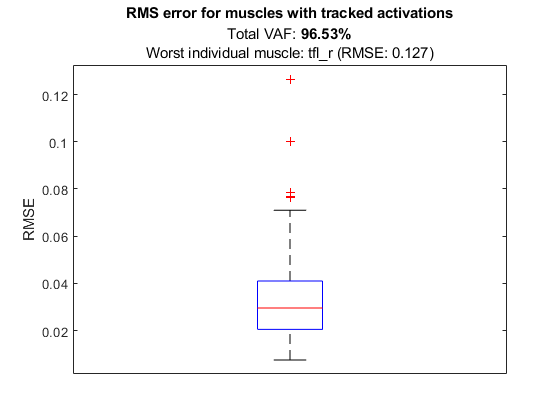

Supplement: Supplement 1 [file media-1.zip › SupplementaryMaterial/NCP/VAF.png]

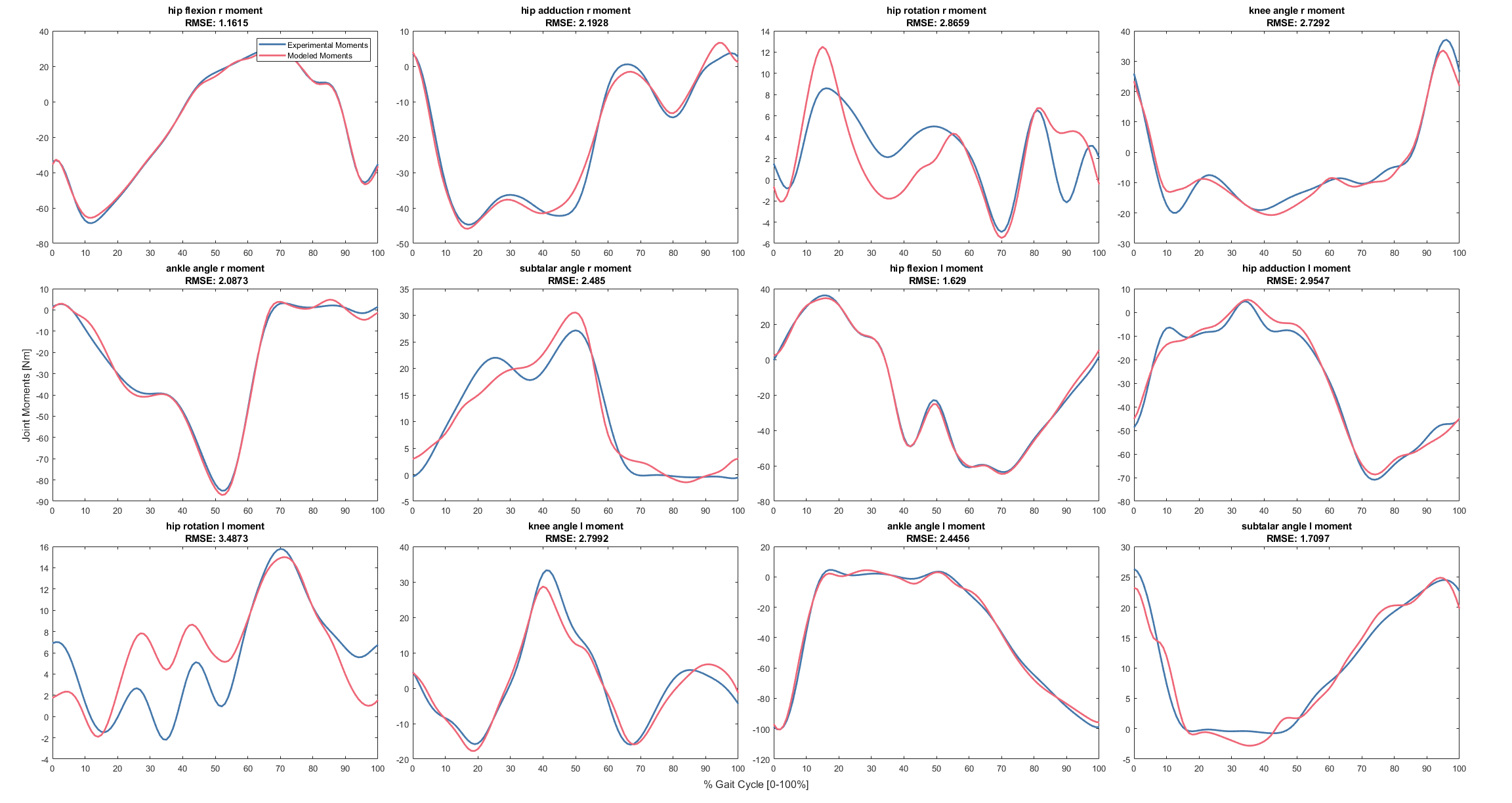

Supplement: Supplement 1 [file media-1.zip › SupplementaryMaterial/NCP/jointMoments.png]

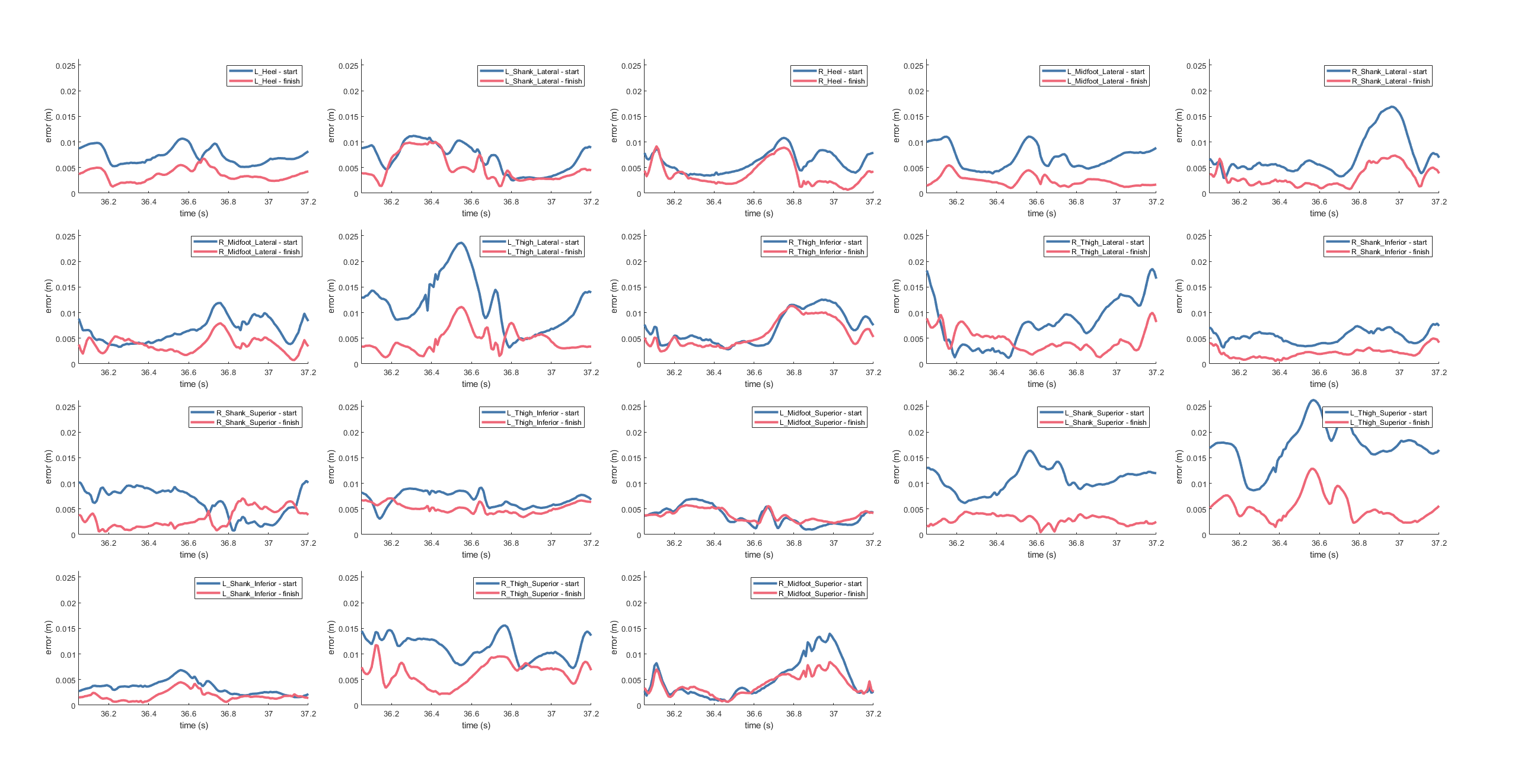

Supplement: Supplement 1 [file media-1.zip › SupplementaryMaterial/JMP/markerErrors.png]

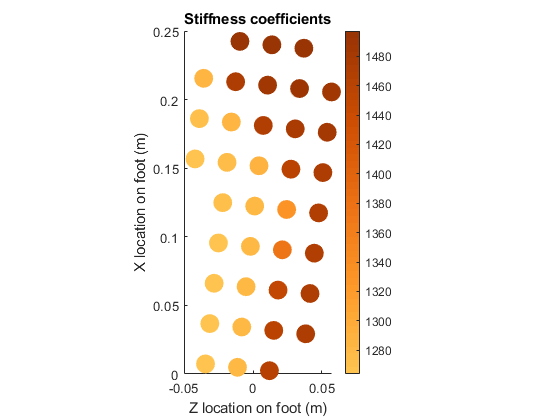

Supplement: Supplement 1 [file media-1.zip › SupplementaryMaterial/GCP/stiffnessCoefficients.png]

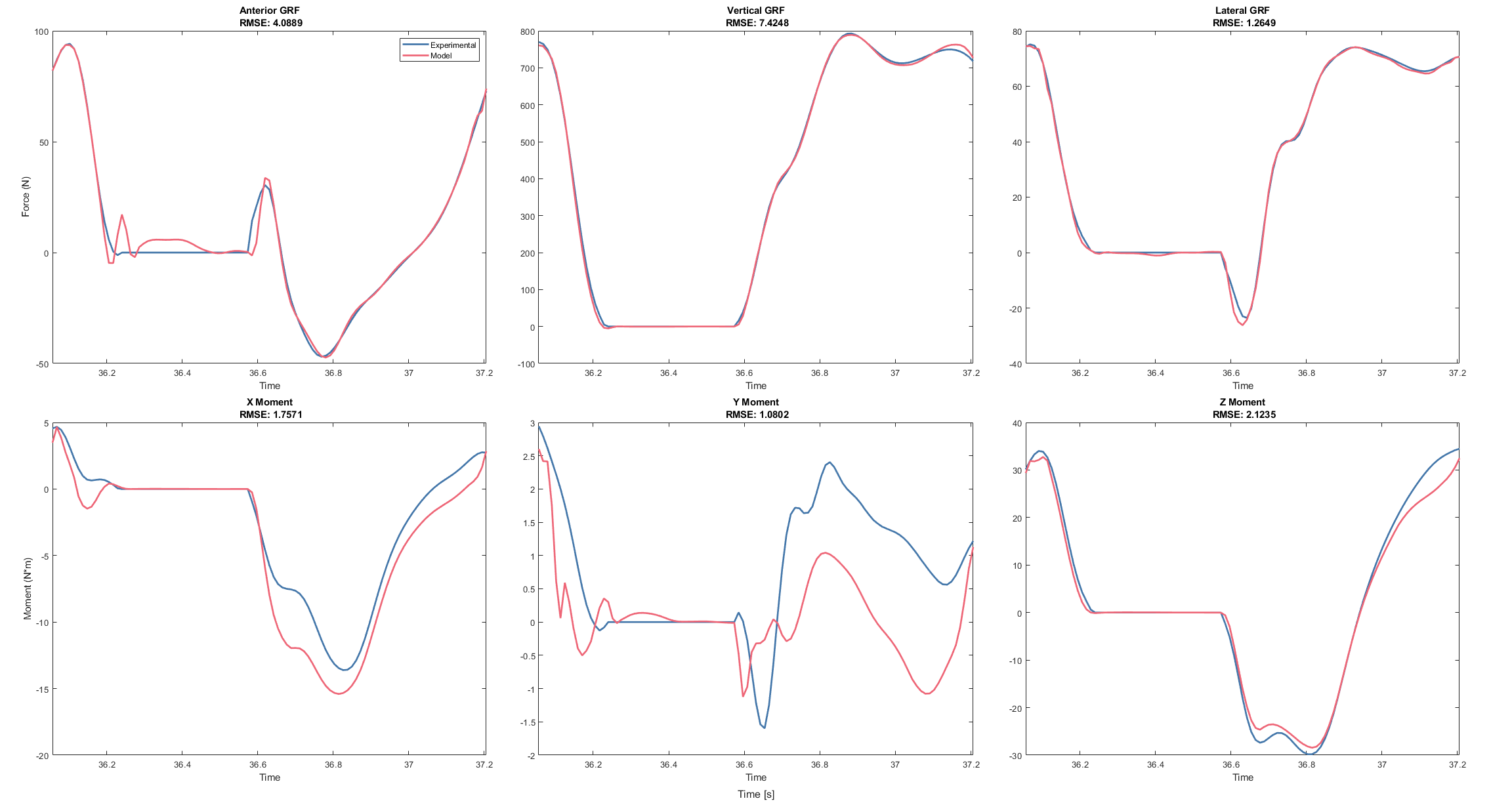

Supplement: Supplement 1 [file media-1.zip › SupplementaryMaterial/GCP/foot2GroundReactions.png]

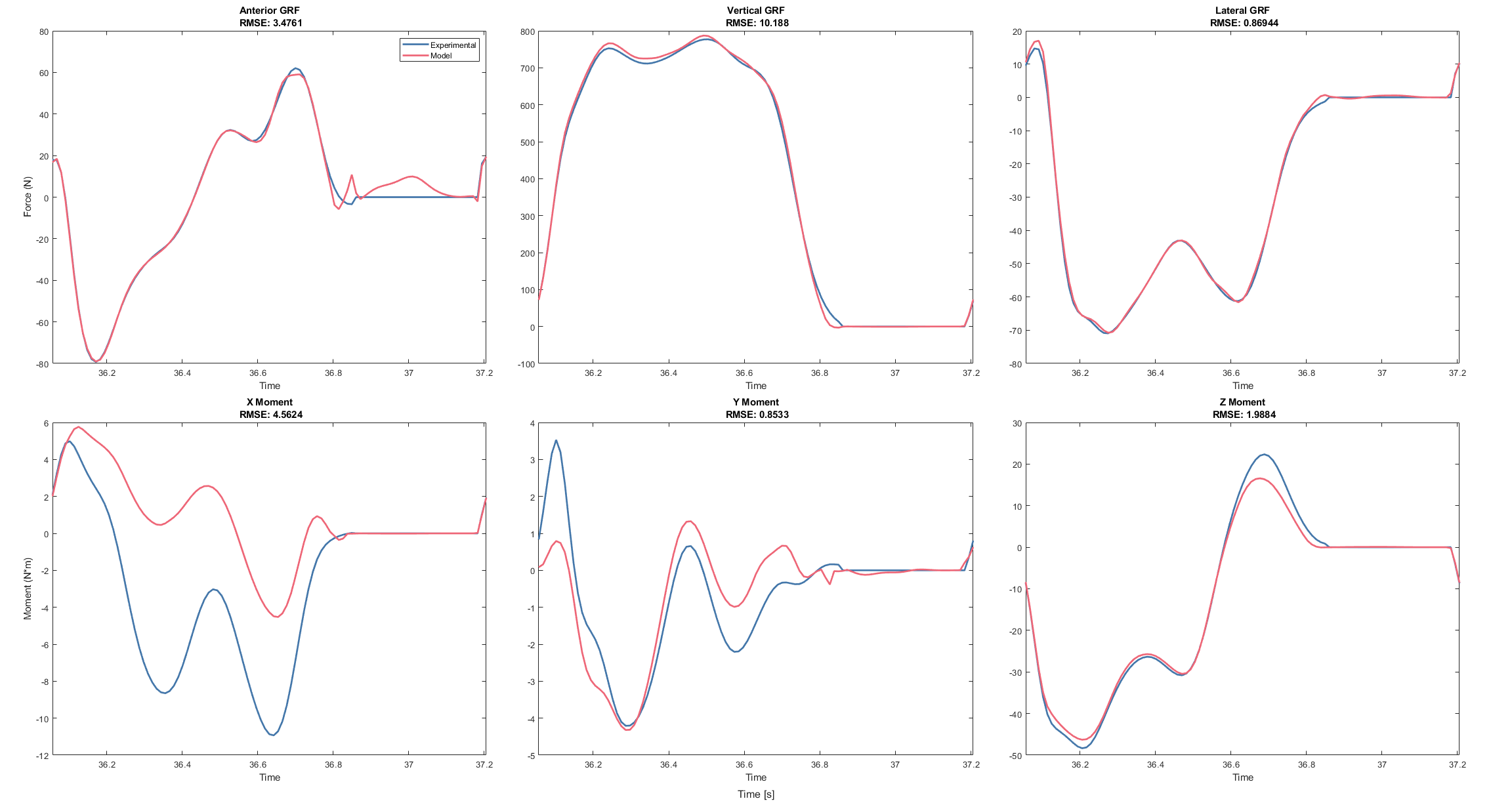

Supplement: Supplement 1 [file media-1.zip › SupplementaryMaterial/GCP/foot1GroundReactions.png]

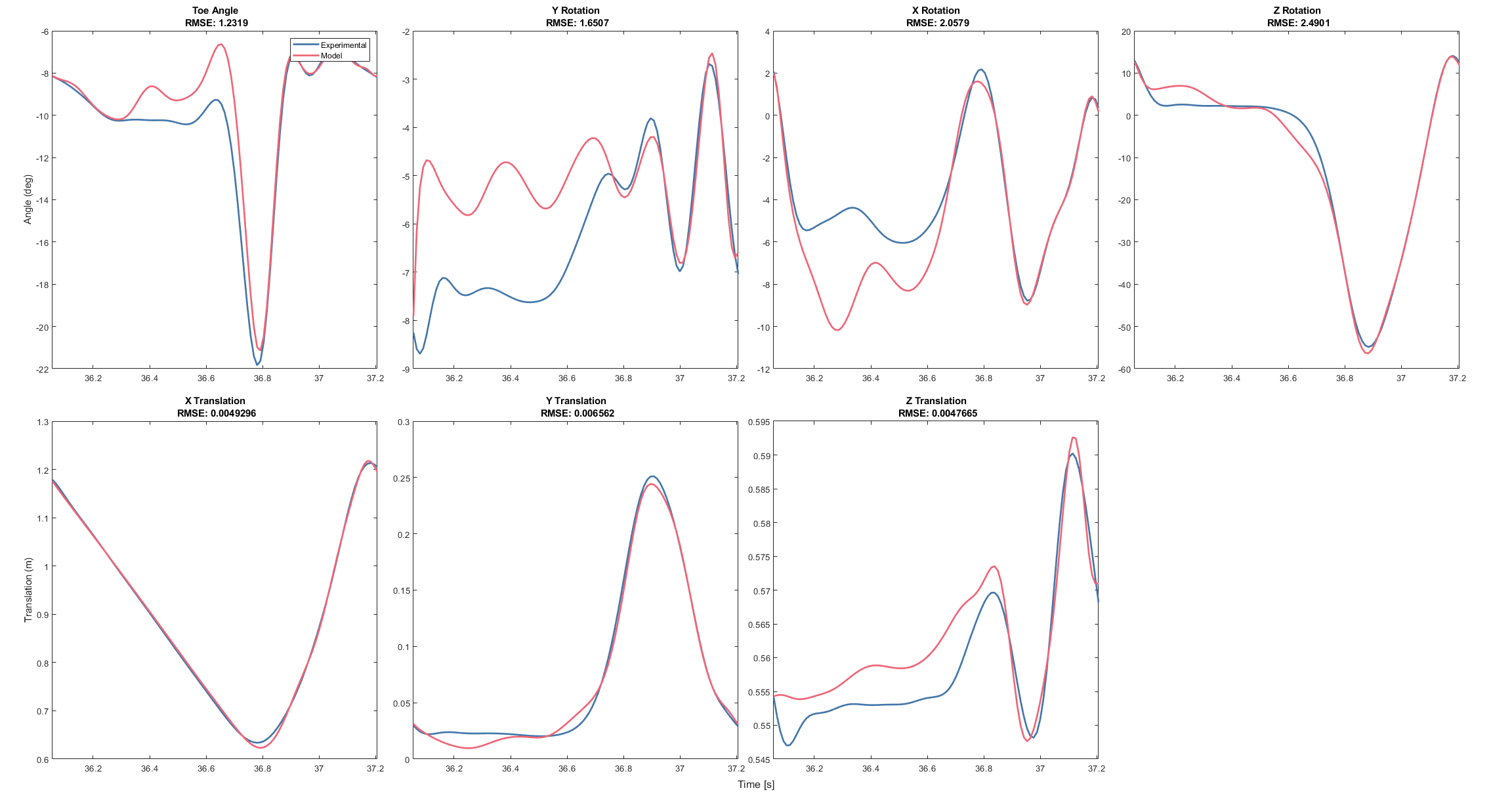

Supplement: Supplement 1 [file media-1.zip › SupplementaryMaterial/GCP/foot1Kinematics.png]

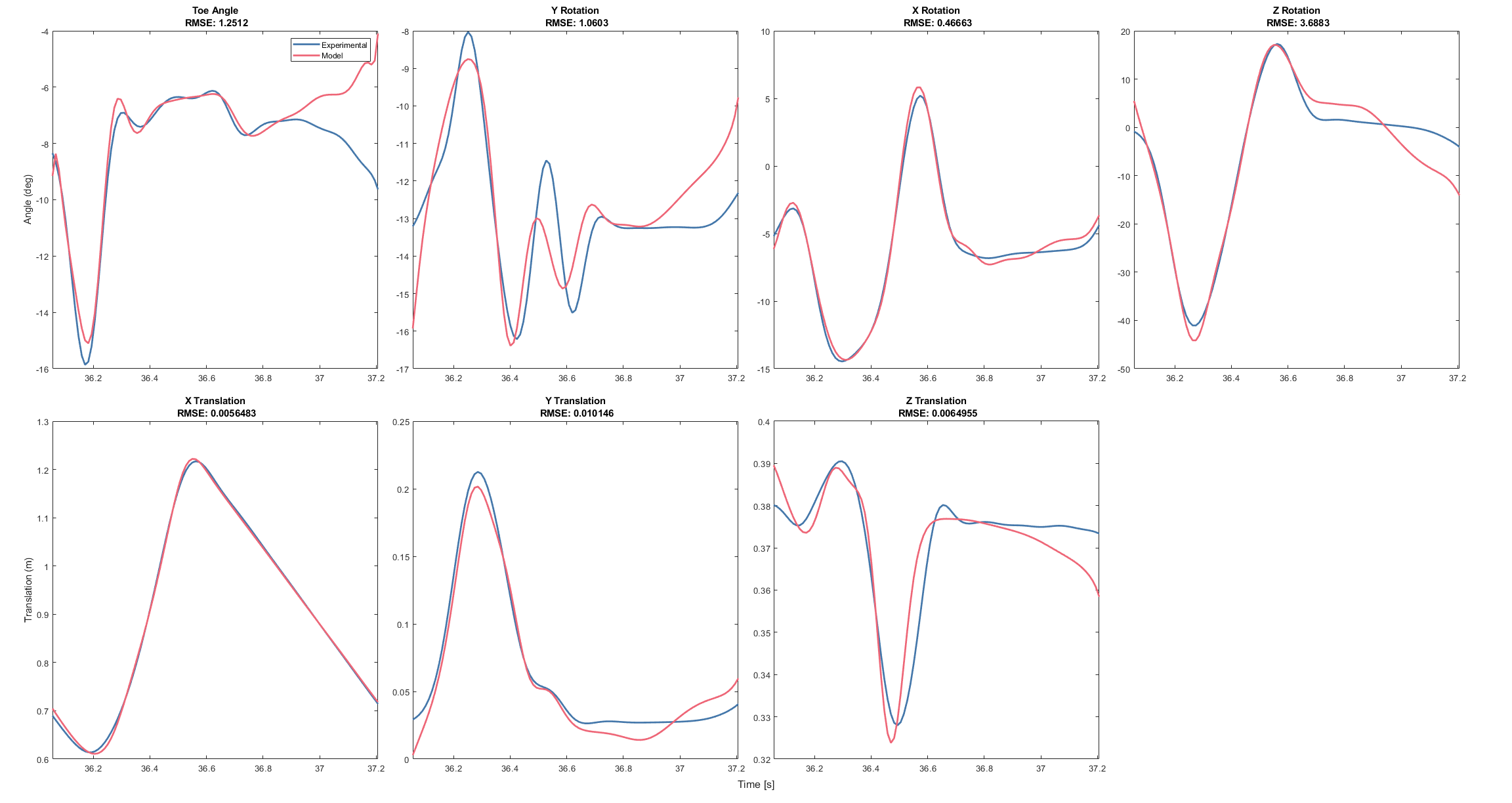

Supplement: Supplement 1 [file media-1.zip › SupplementaryMaterial/GCP/foot2Kinematics.png]

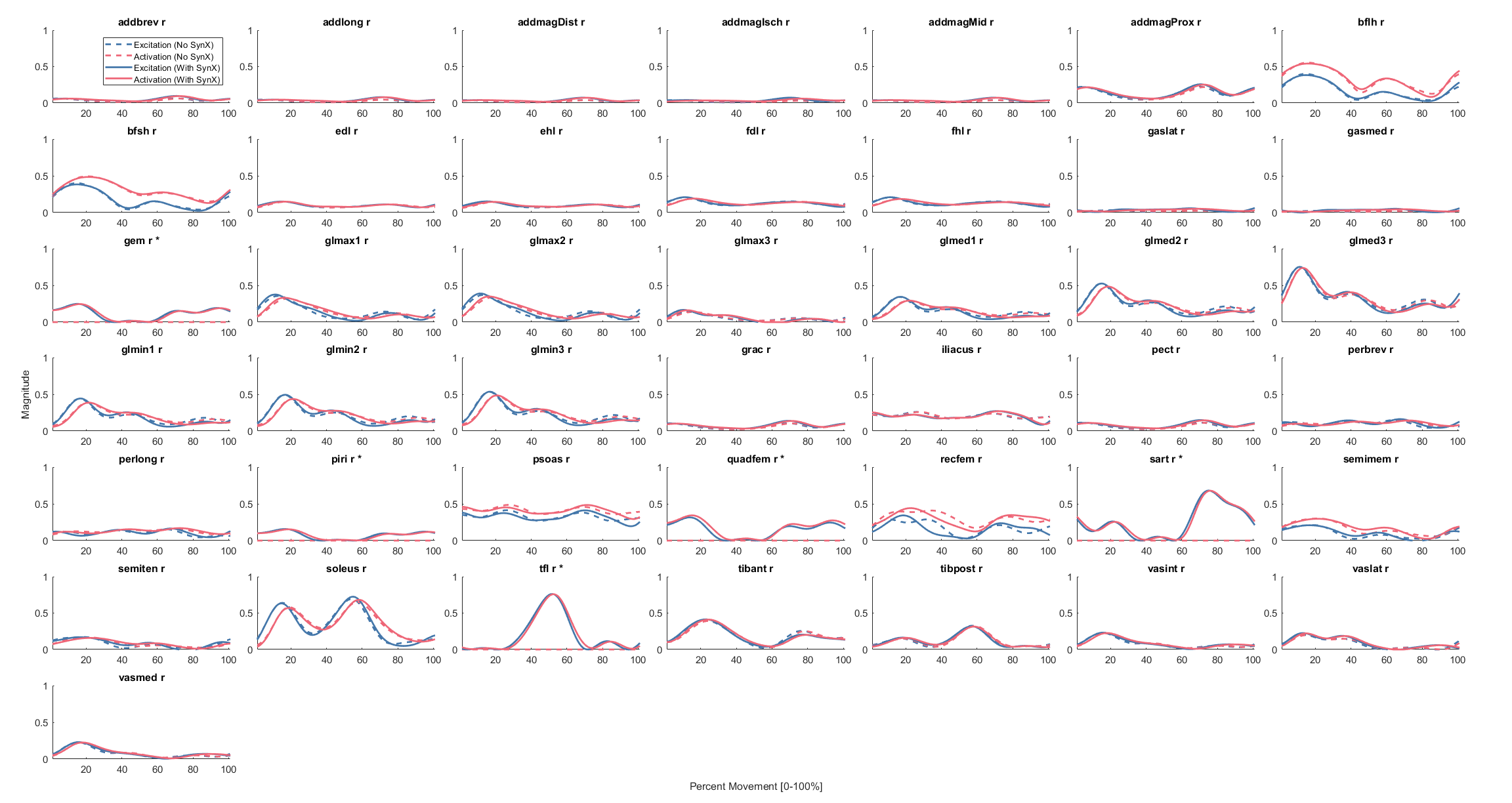

Supplement: Supplement 1 [file media-1.zip › SupplementaryMaterial/MTP/rightActivation.png]

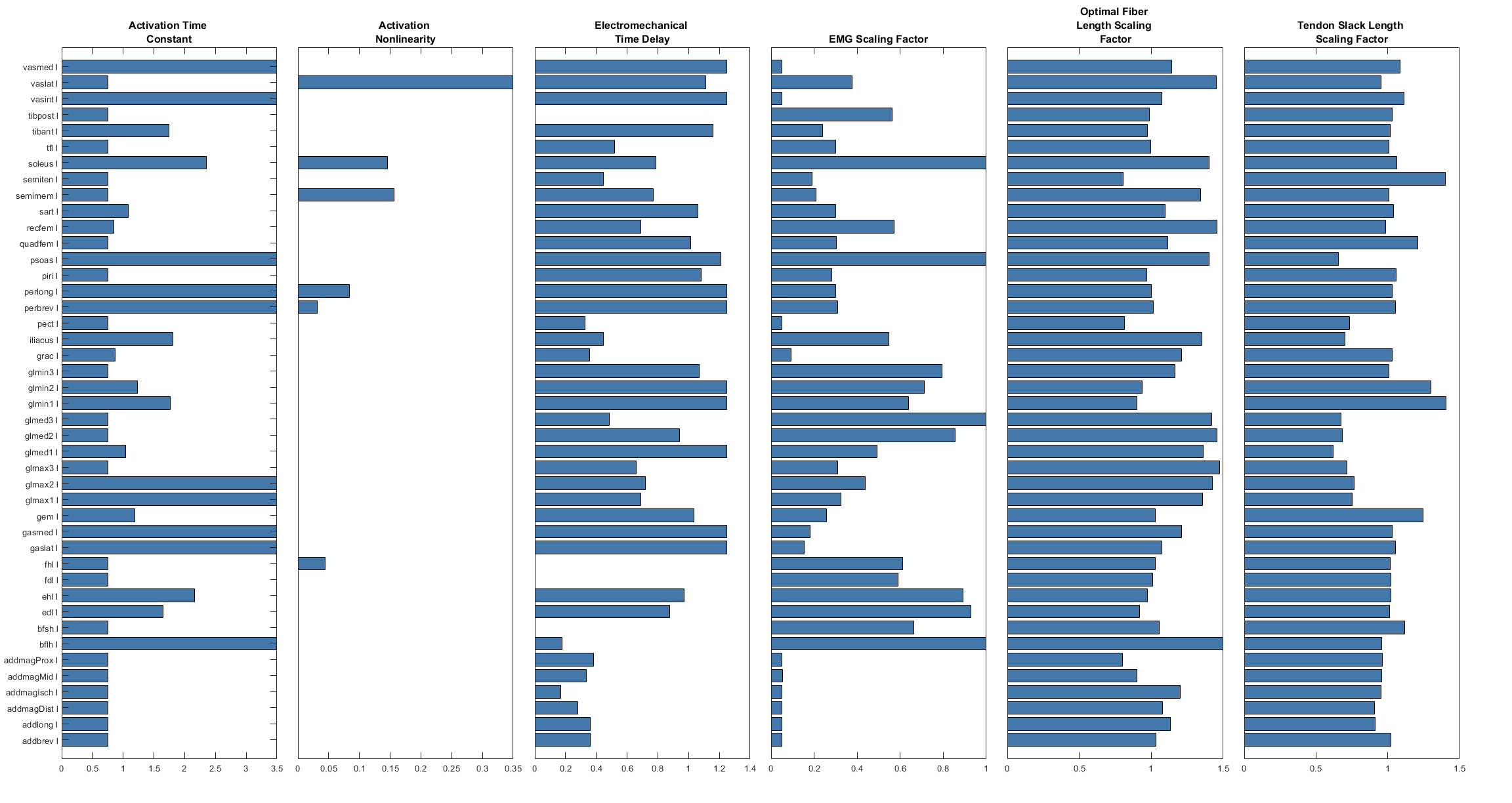

Supplement: Supplement 1 [file media-1.zip › SupplementaryMaterial/MTP/leftParameters.png]

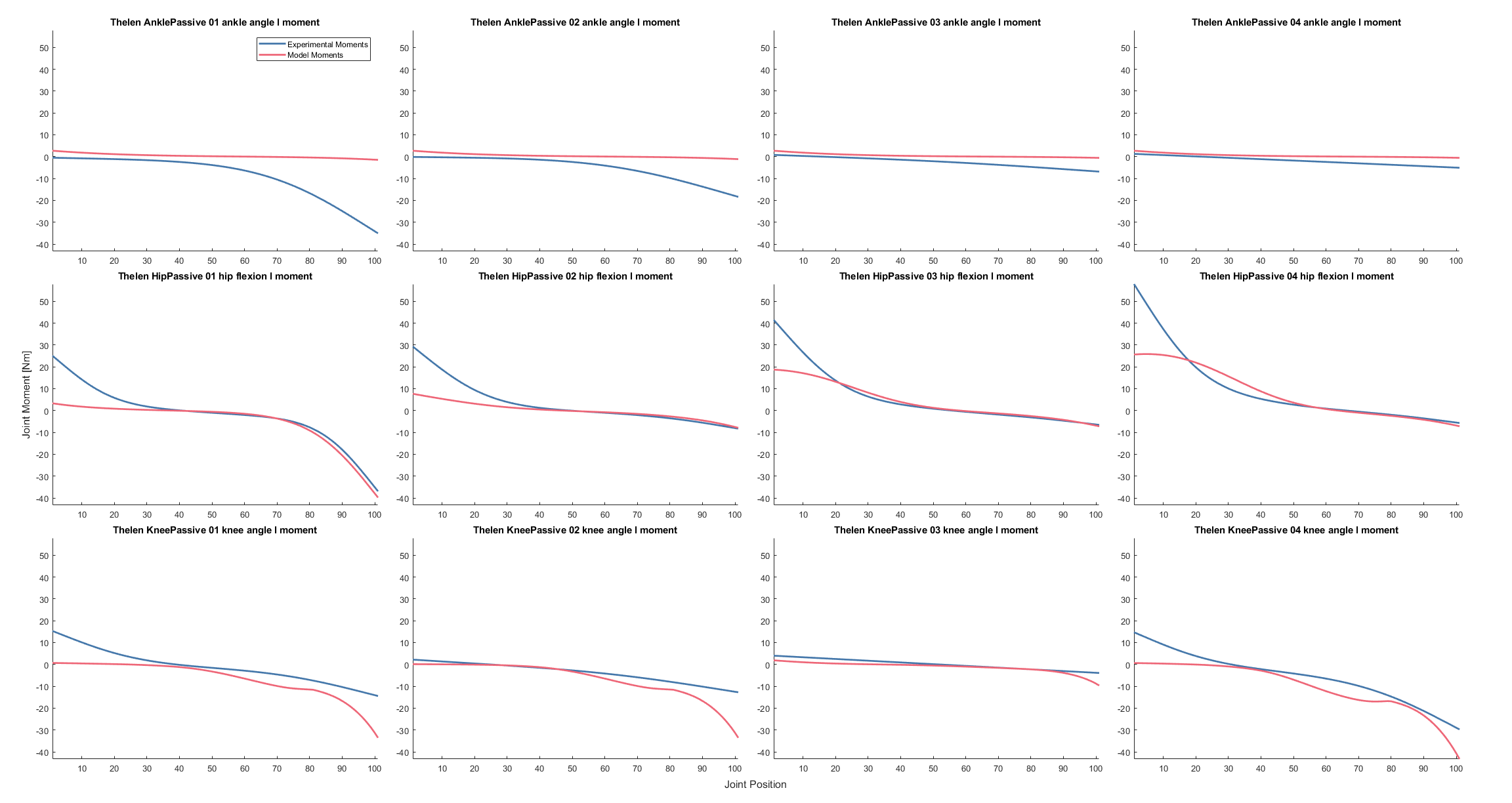

Supplement: Supplement 1 [file media-1.zip › SupplementaryMaterial/MTP/leftPassiveMoment.png]

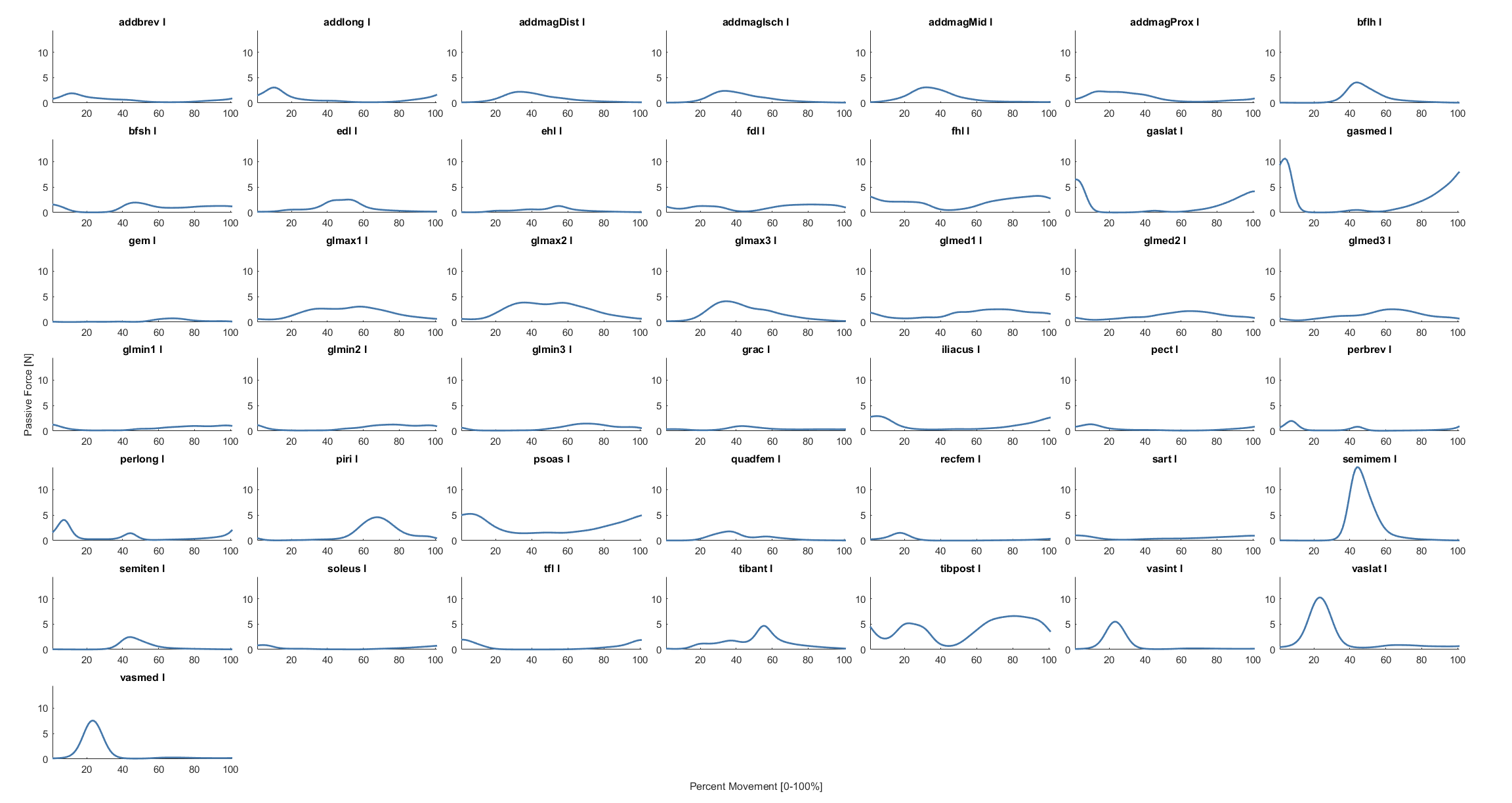

Supplement: Supplement 1 [file media-1.zip › SupplementaryMaterial/MTP/leftPassiveForce.png]

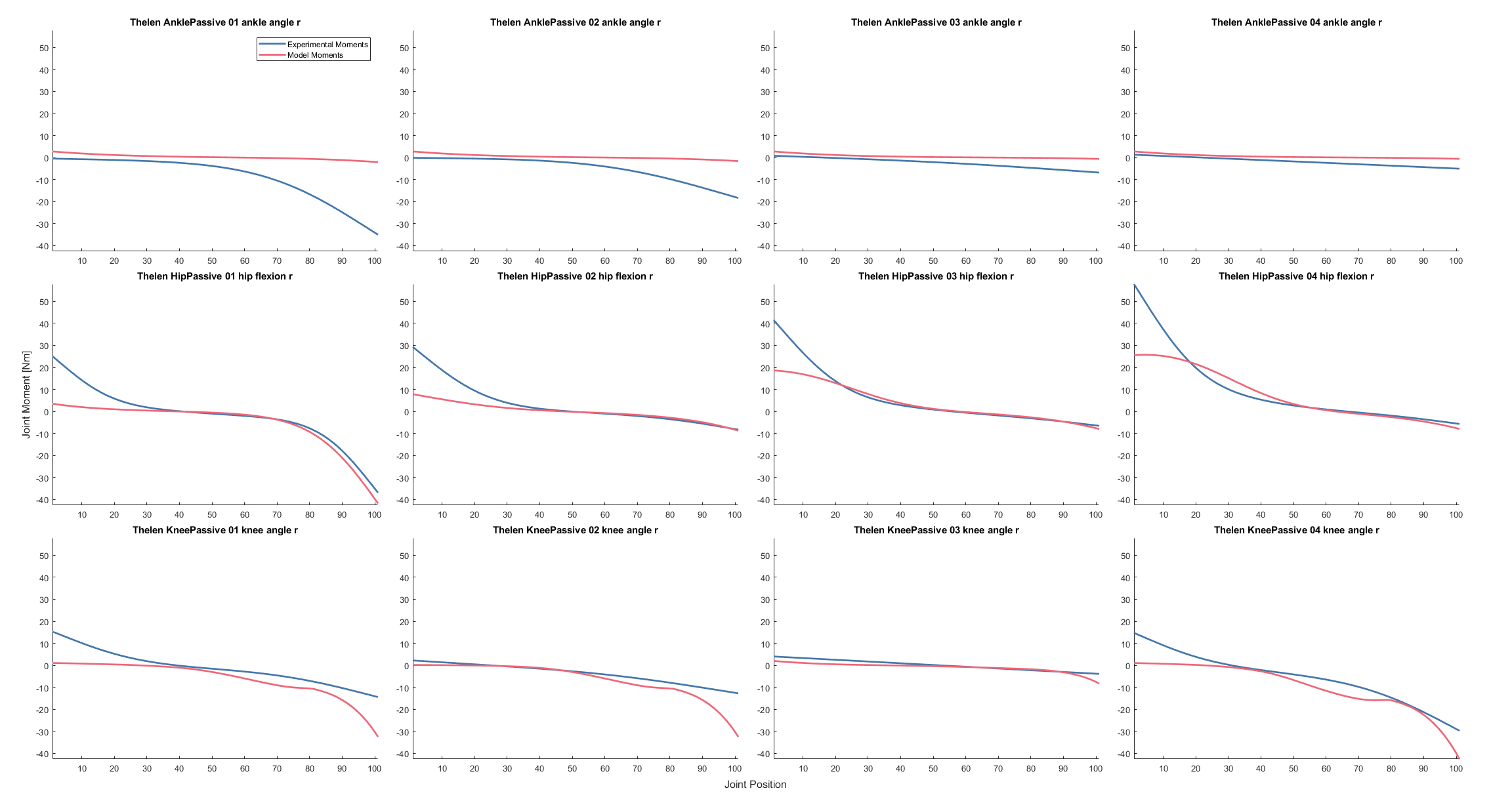

Supplement: Supplement 1 [file media-1.zip › SupplementaryMaterial/MTP/rightPassiveMoment.png]

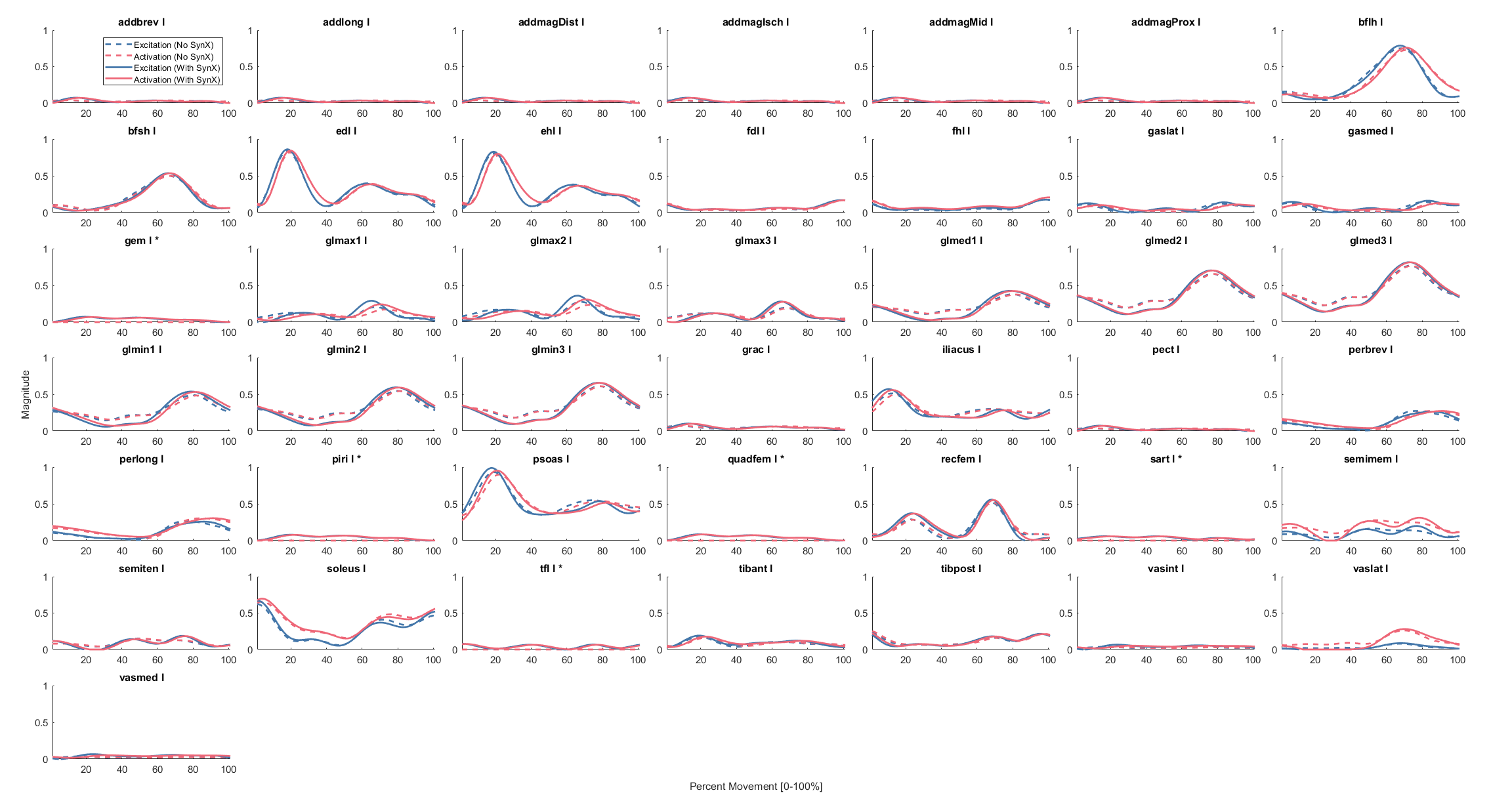

Supplement: Supplement 1 [file media-1.zip › SupplementaryMaterial/MTP/leftActivation.png]

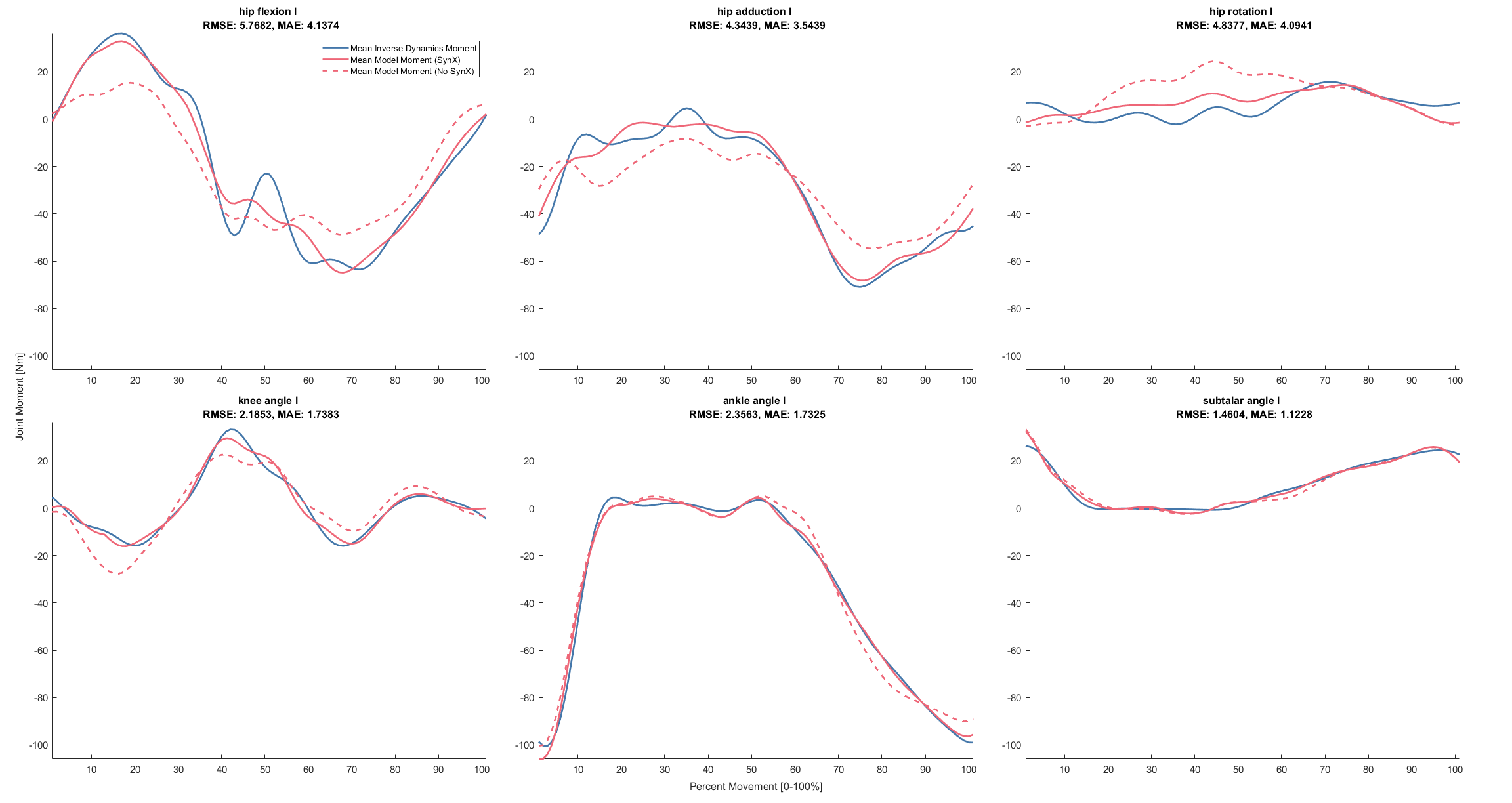

Supplement: Supplement 1 [file media-1.zip › SupplementaryMaterial/MTP/leftMoment.png]

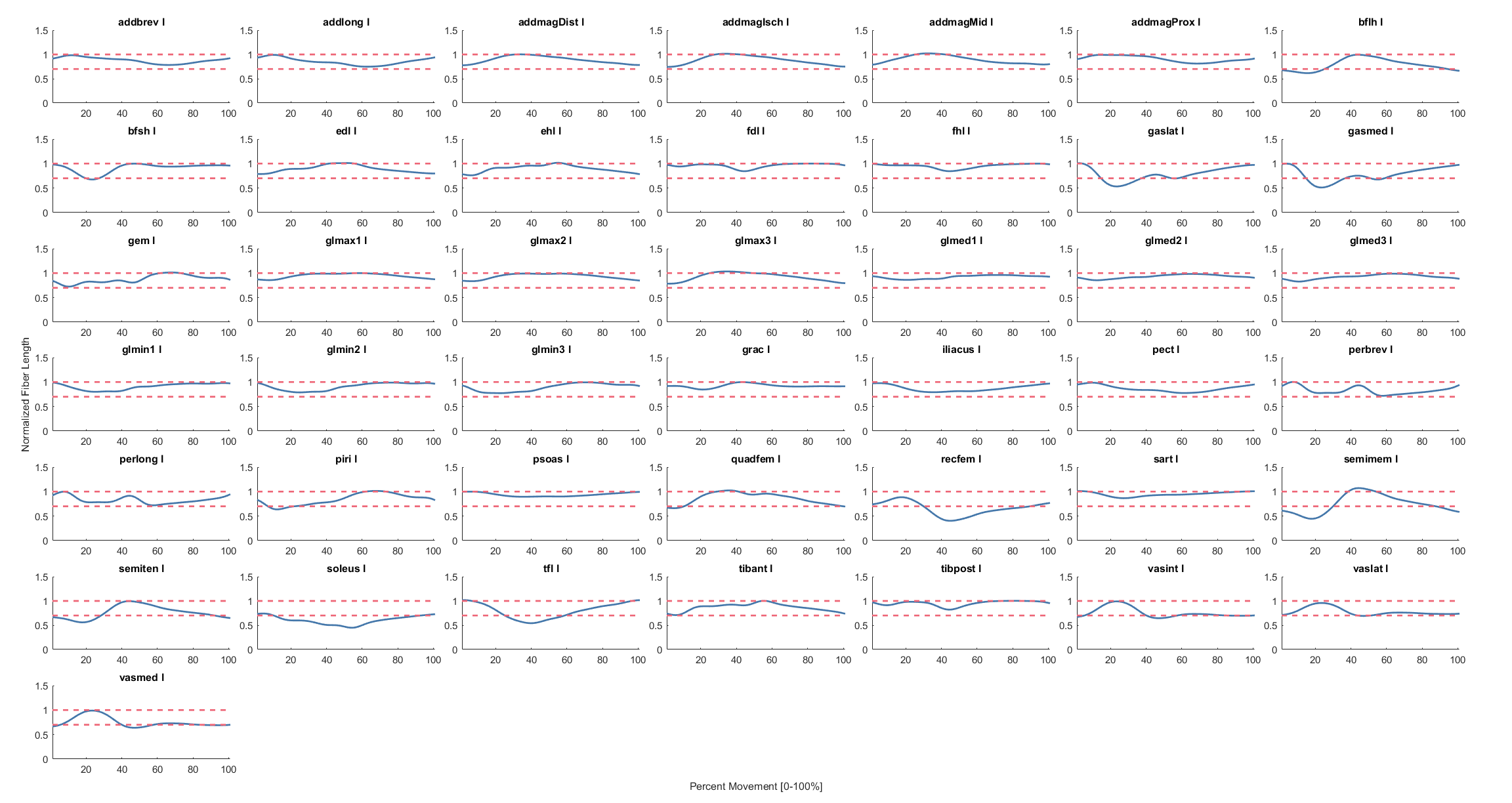

Supplement: Supplement 1 [file media-1.zip › SupplementaryMaterial/MTP/leftFiberLength.png]

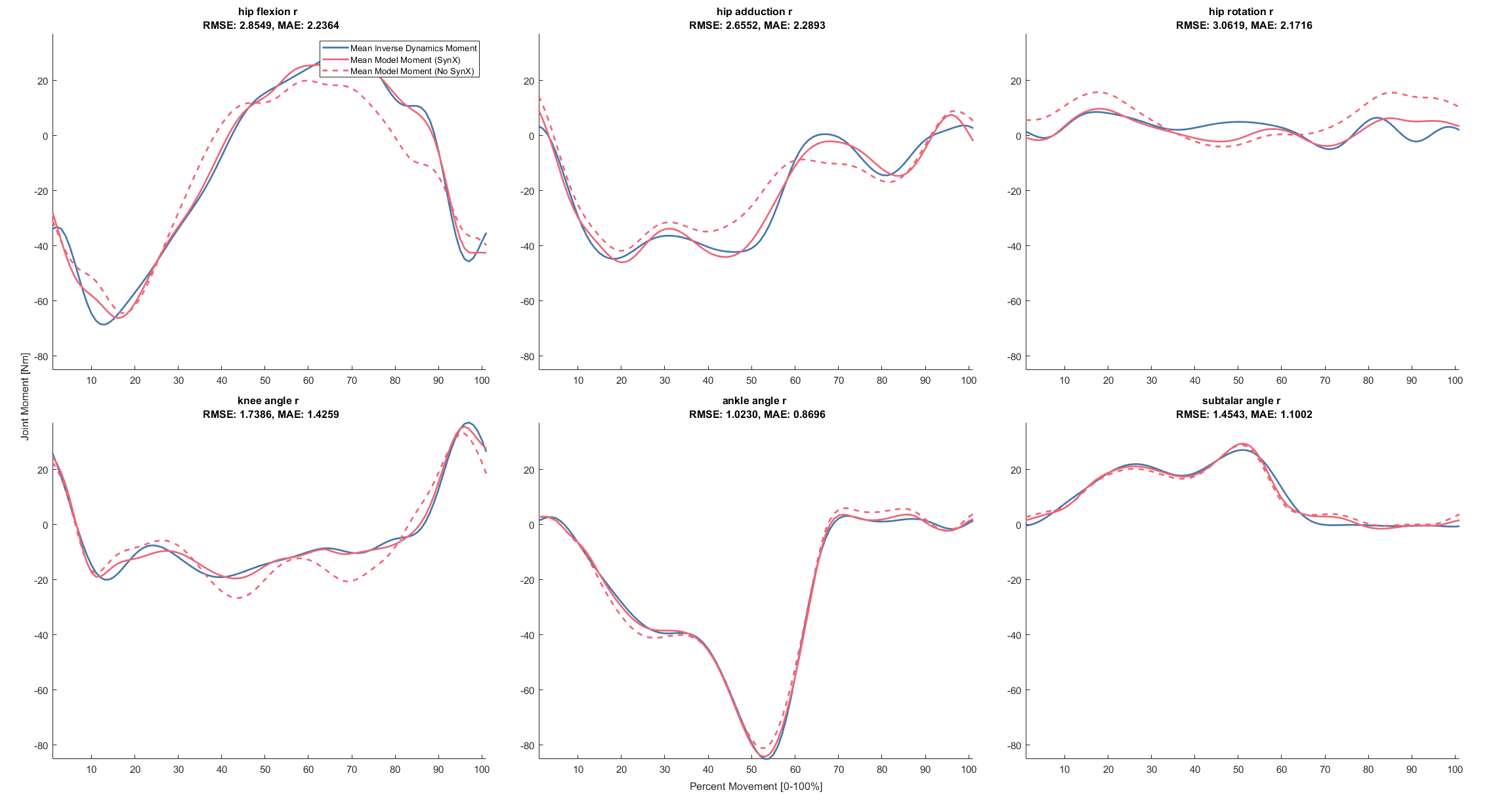

Supplement: Supplement 1 [file media-1.zip › SupplementaryMaterial/MTP/rightMoment.png]

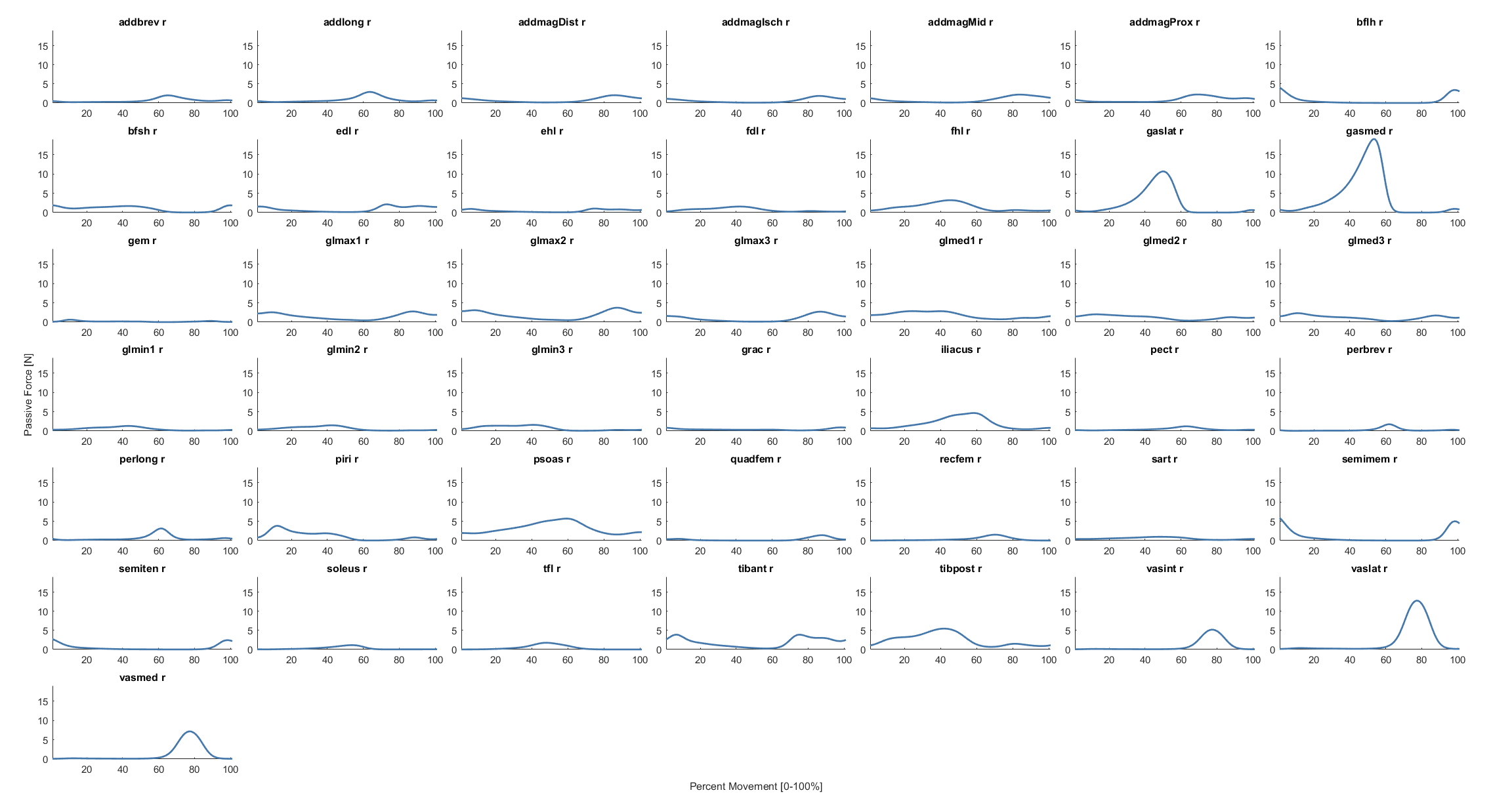

Supplement: Supplement 1 [file media-1.zip › SupplementaryMaterial/MTP/rightPassiveForce.png]

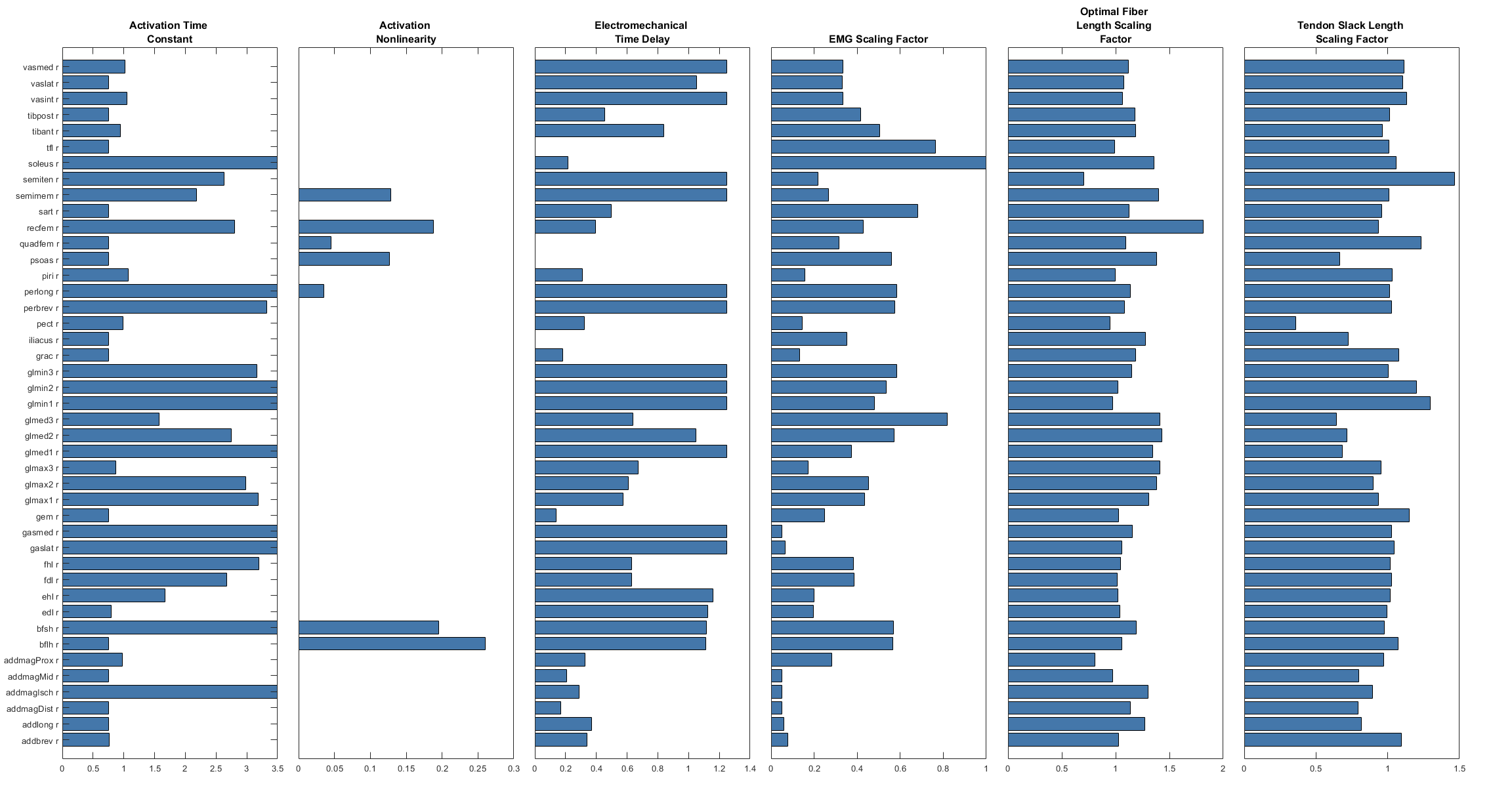

Supplement: Supplement 1 [file media-1.zip › SupplementaryMaterial/MTP/rightParameters.png]

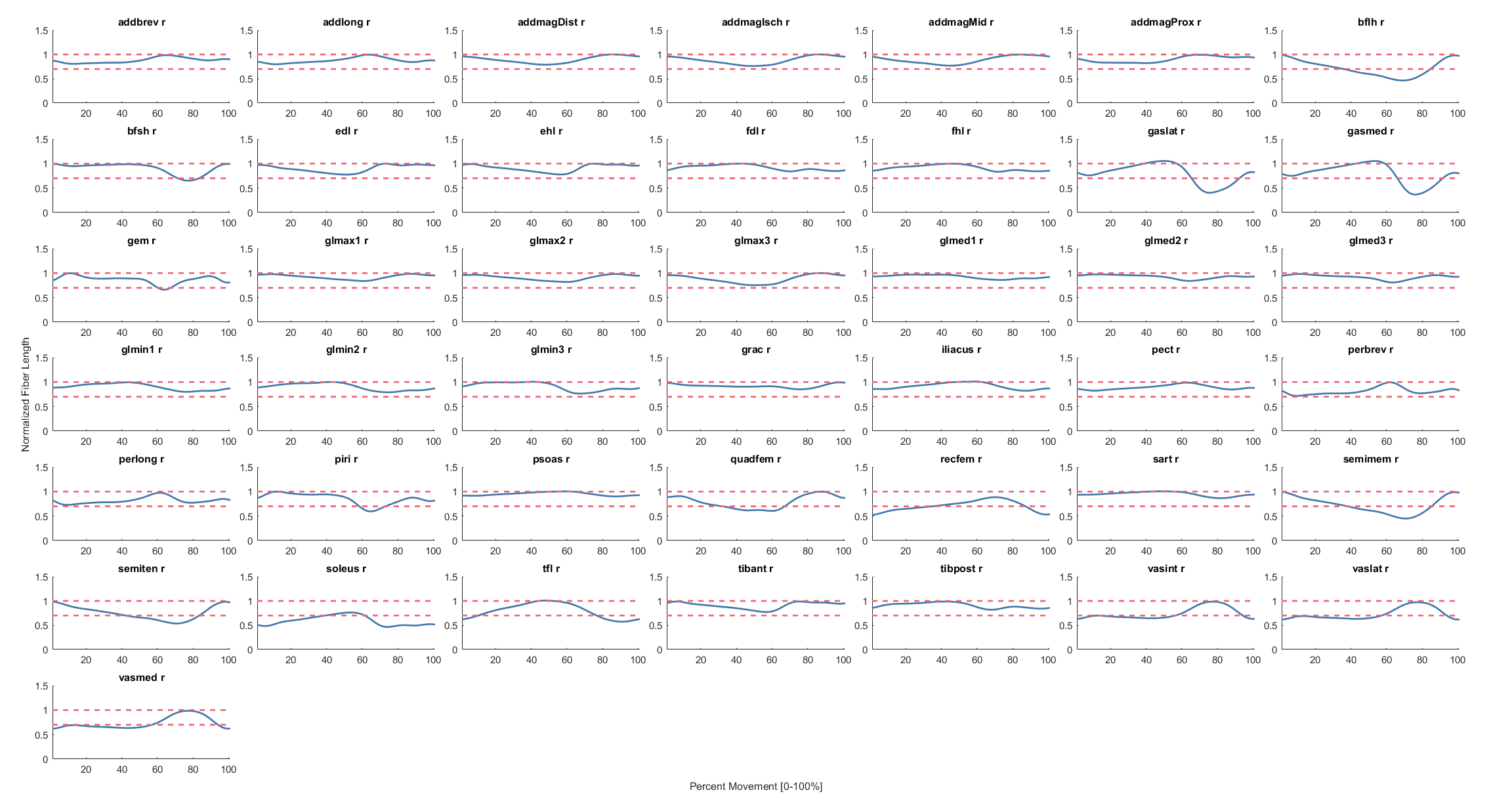

Supplement: Supplement 1 [file media-1.zip › SupplementaryMaterial/MTP/rightFiberLength.png]

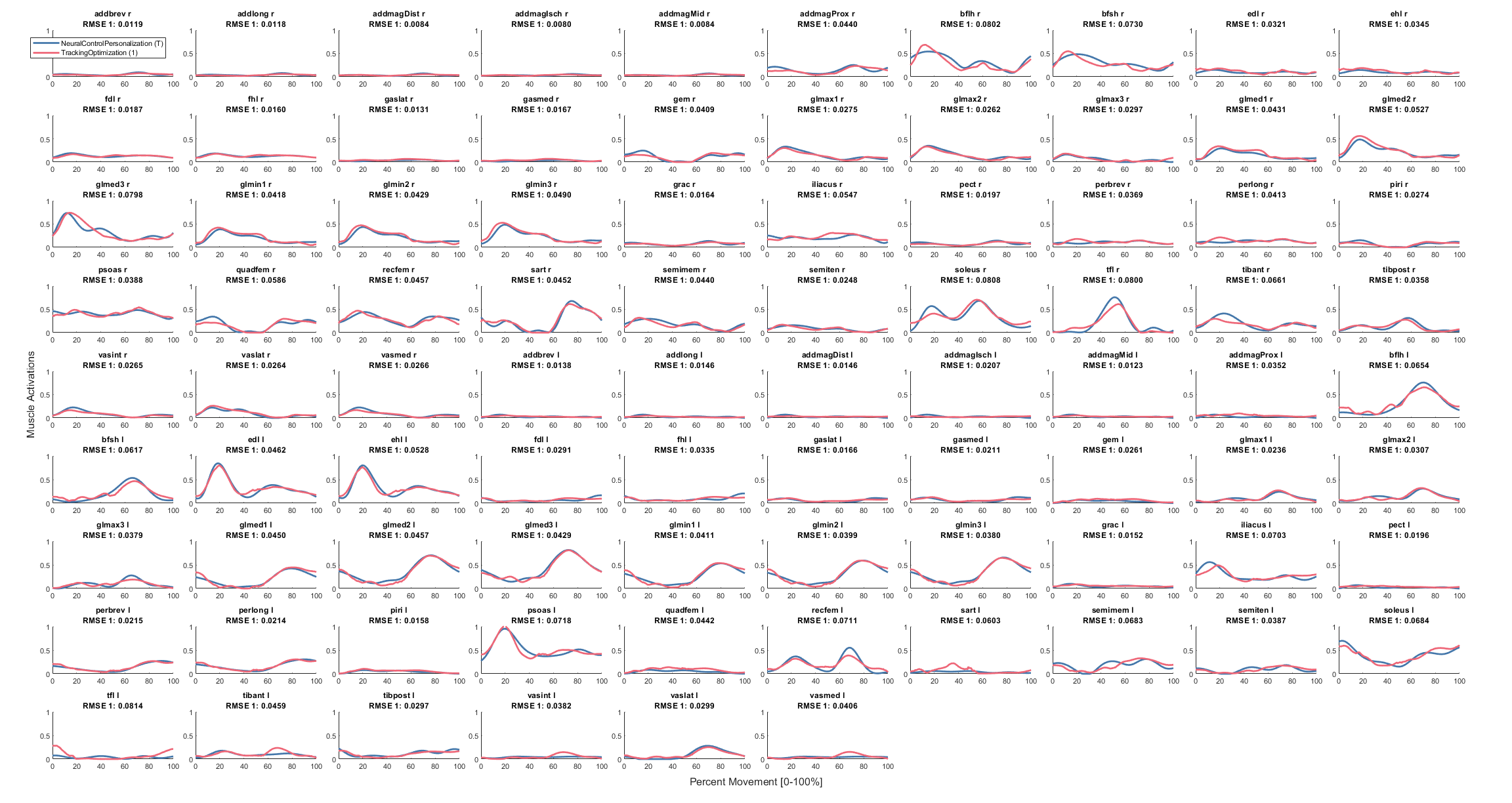

Supplement: Supplement 1 [file media-1.zip › SupplementaryMaterial/TO/activations.png]

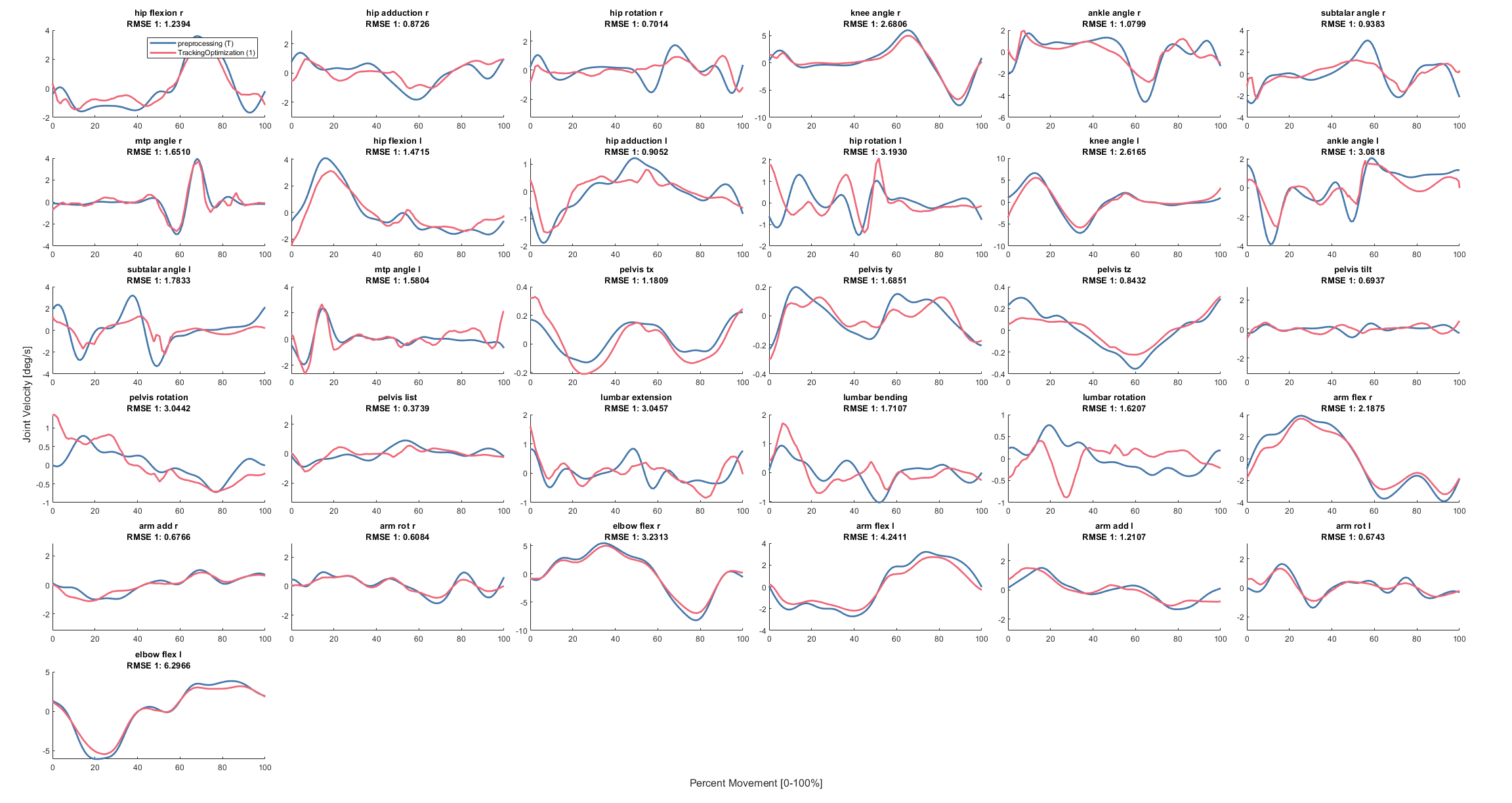

Supplement: Supplement 1 [file media-1.zip › SupplementaryMaterial/TO/jointVelocities.png]

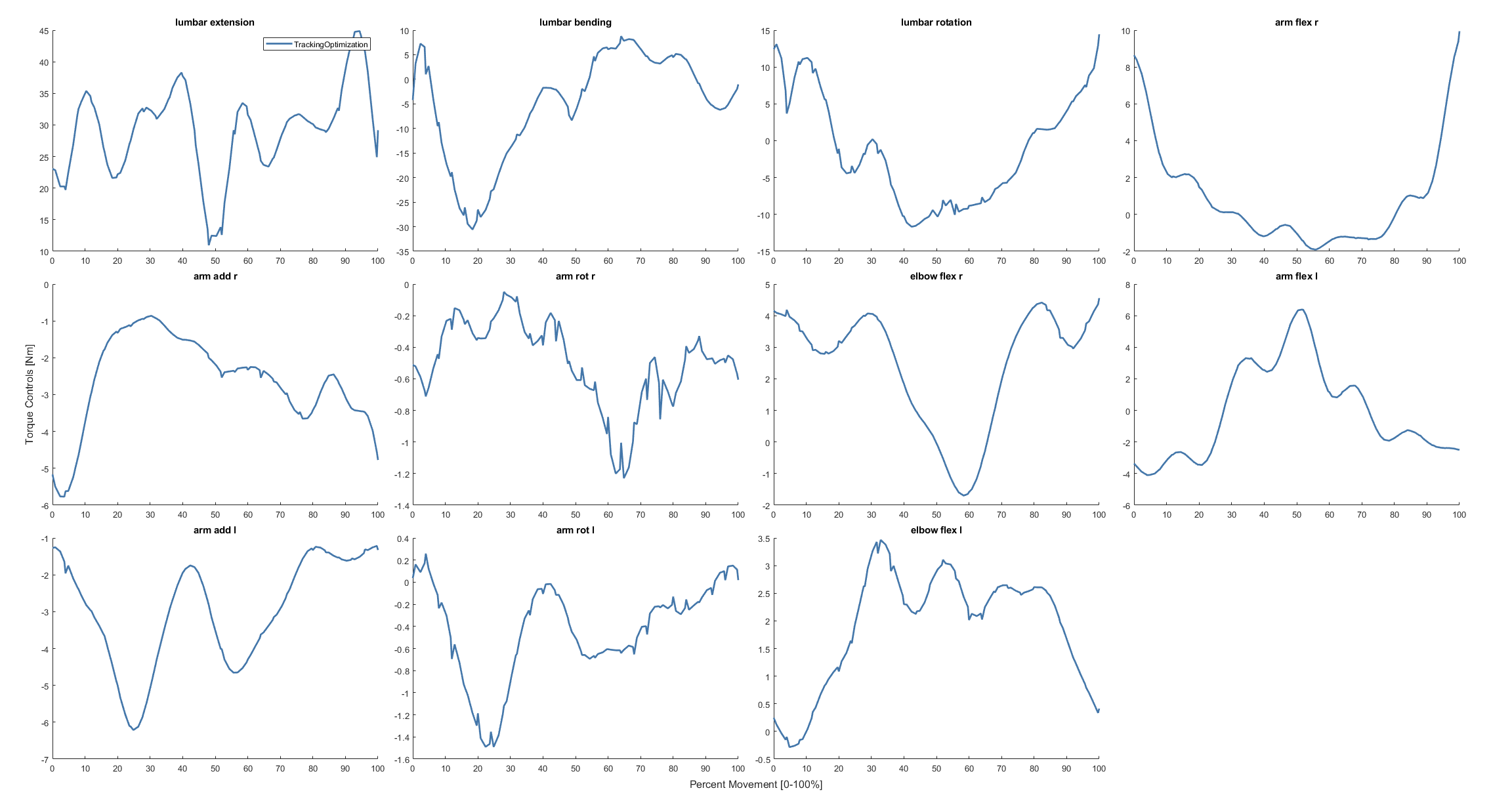

Supplement: Supplement 1 [file media-1.zip › SupplementaryMaterial/TO/torqueControls.png]

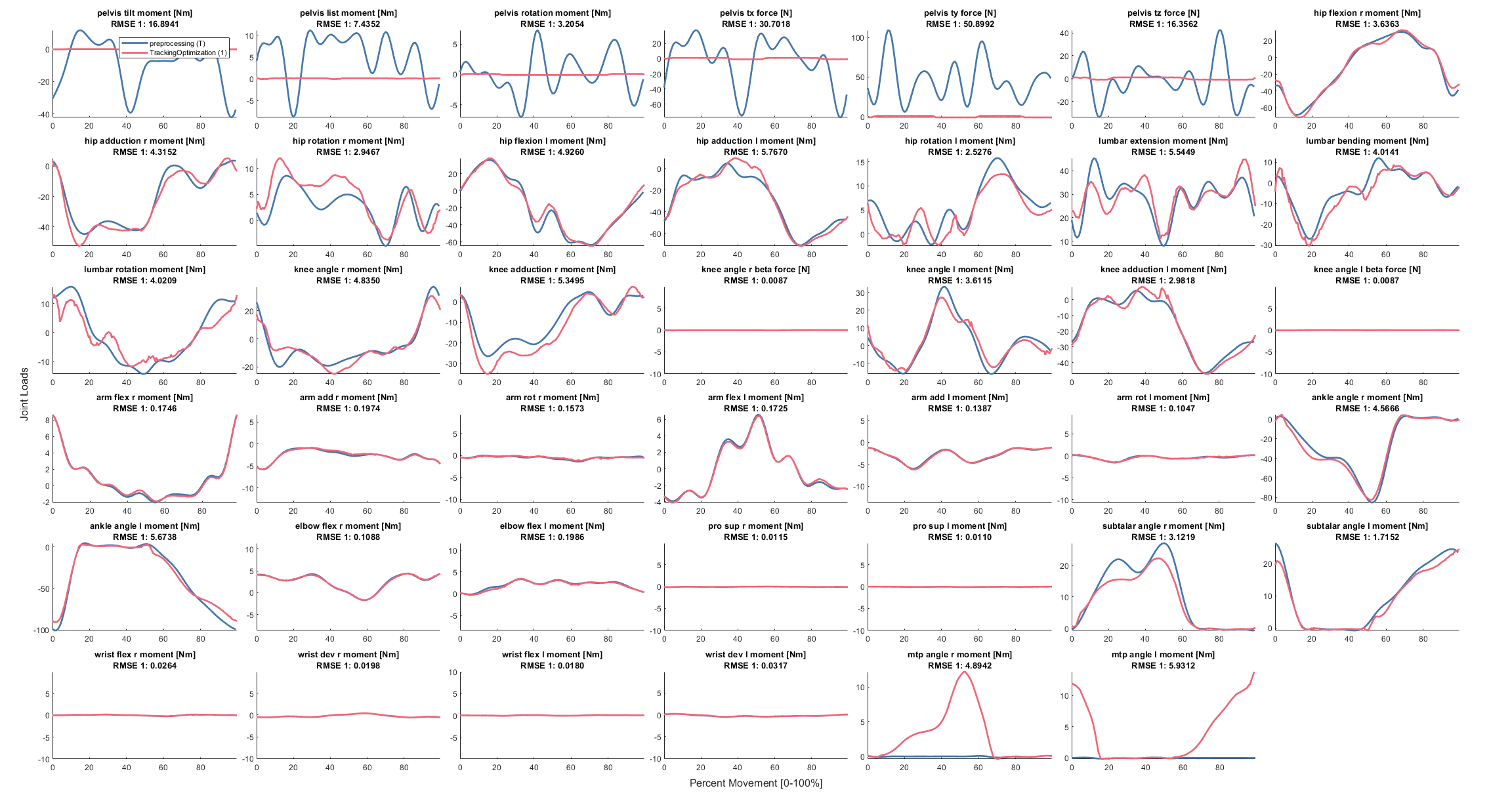

Supplement: Supplement 1 [file media-1.zip › SupplementaryMaterial/TO/jointLoads.png]

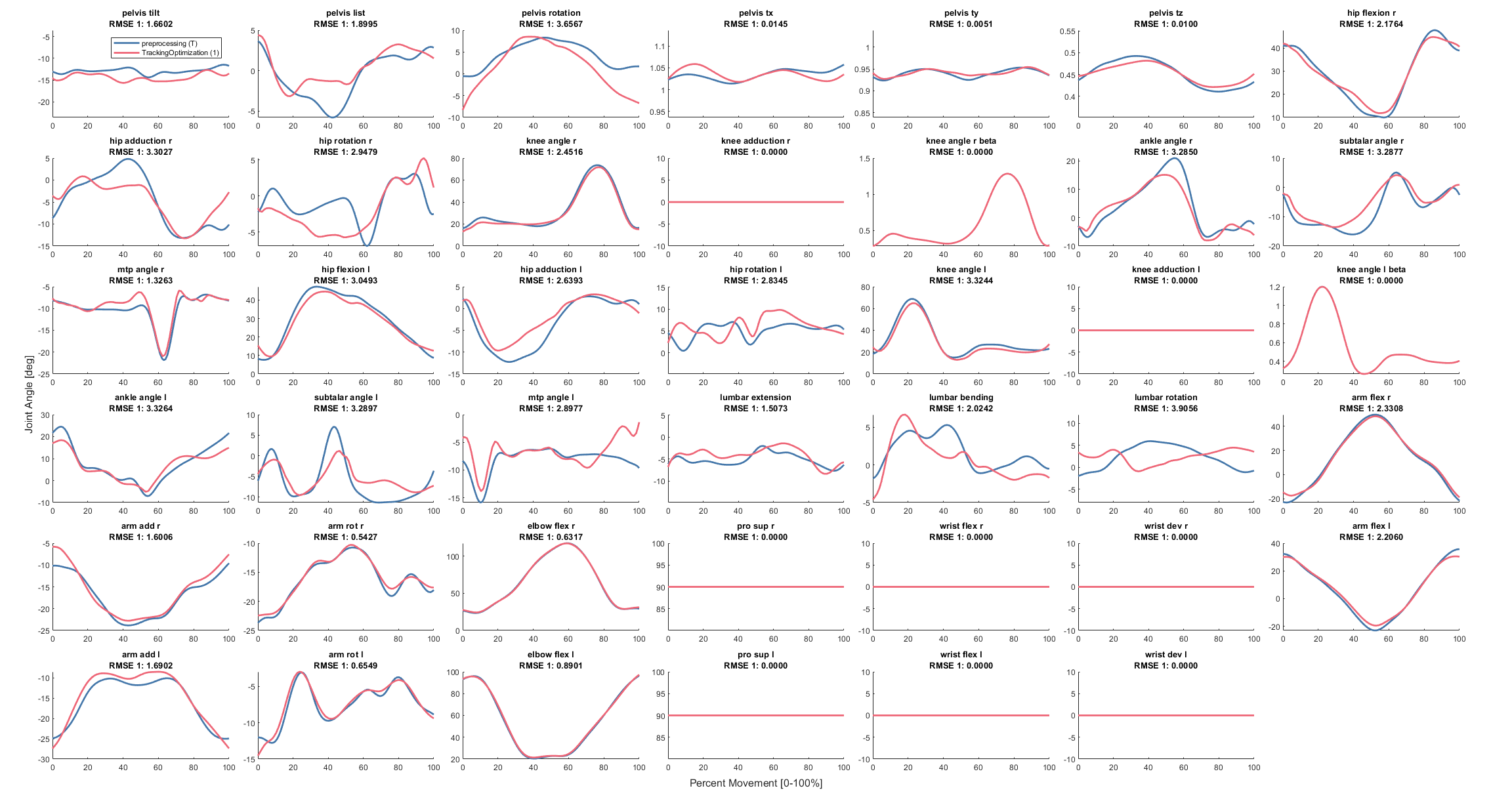

Supplement: Supplement 1 [file media-1.zip › SupplementaryMaterial/TO/jointAngles.png]

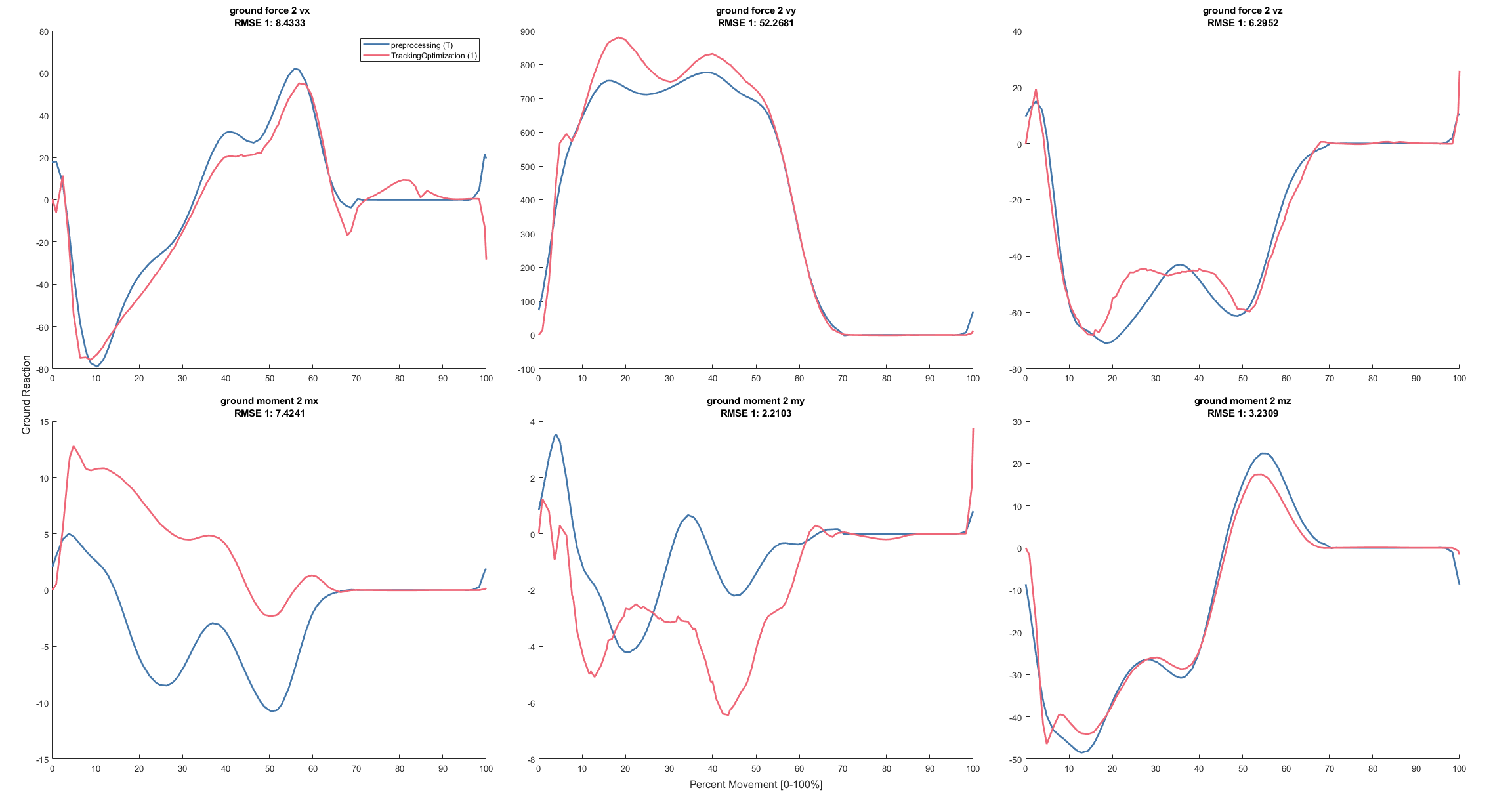

Supplement: Supplement 1 [file media-1.zip › SupplementaryMaterial/TO/foot2GroundReactions.png]

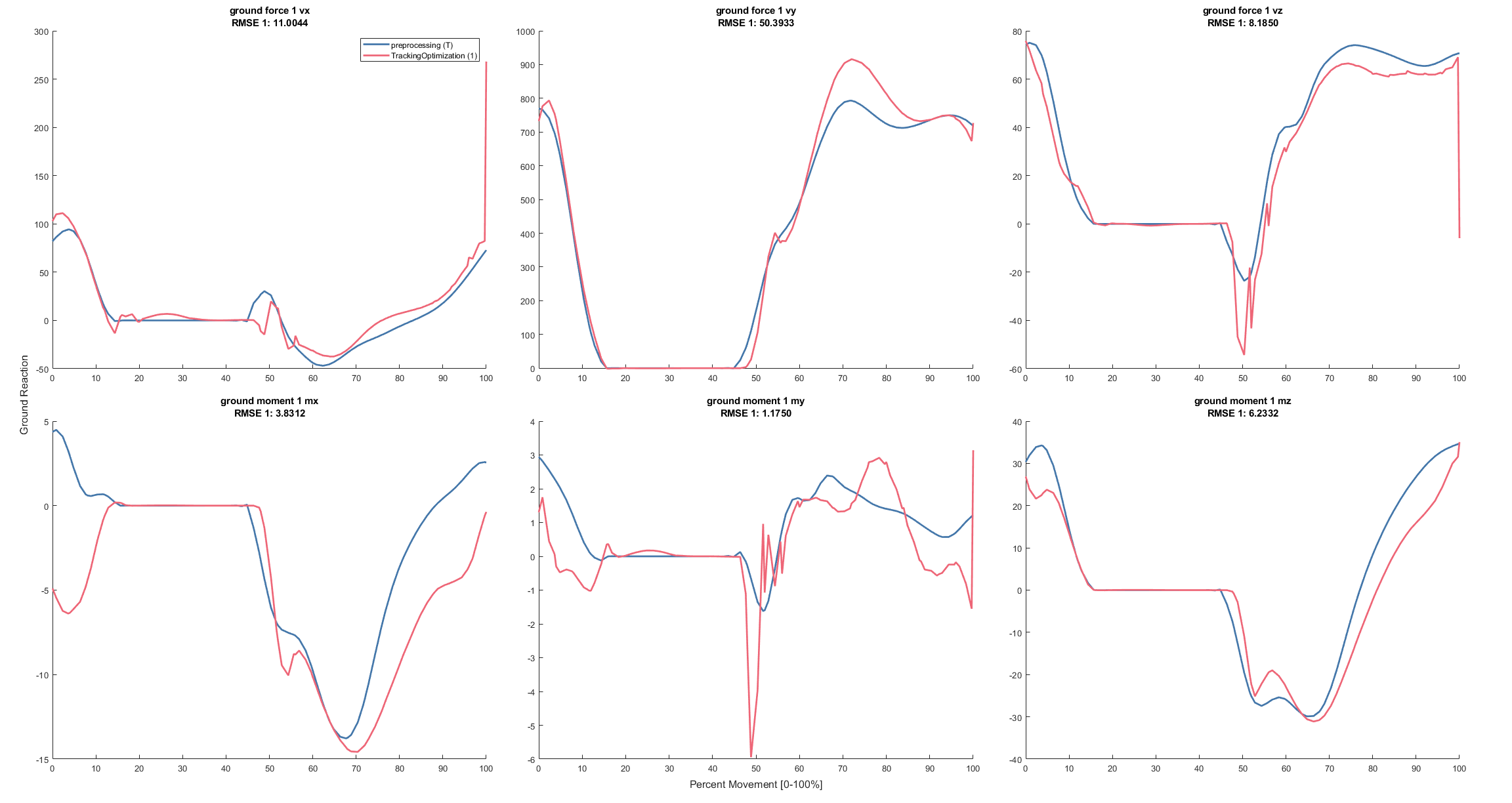

Supplement: Supplement 1 [file media-1.zip › SupplementaryMaterial/TO/foot1GroundReactions.png]

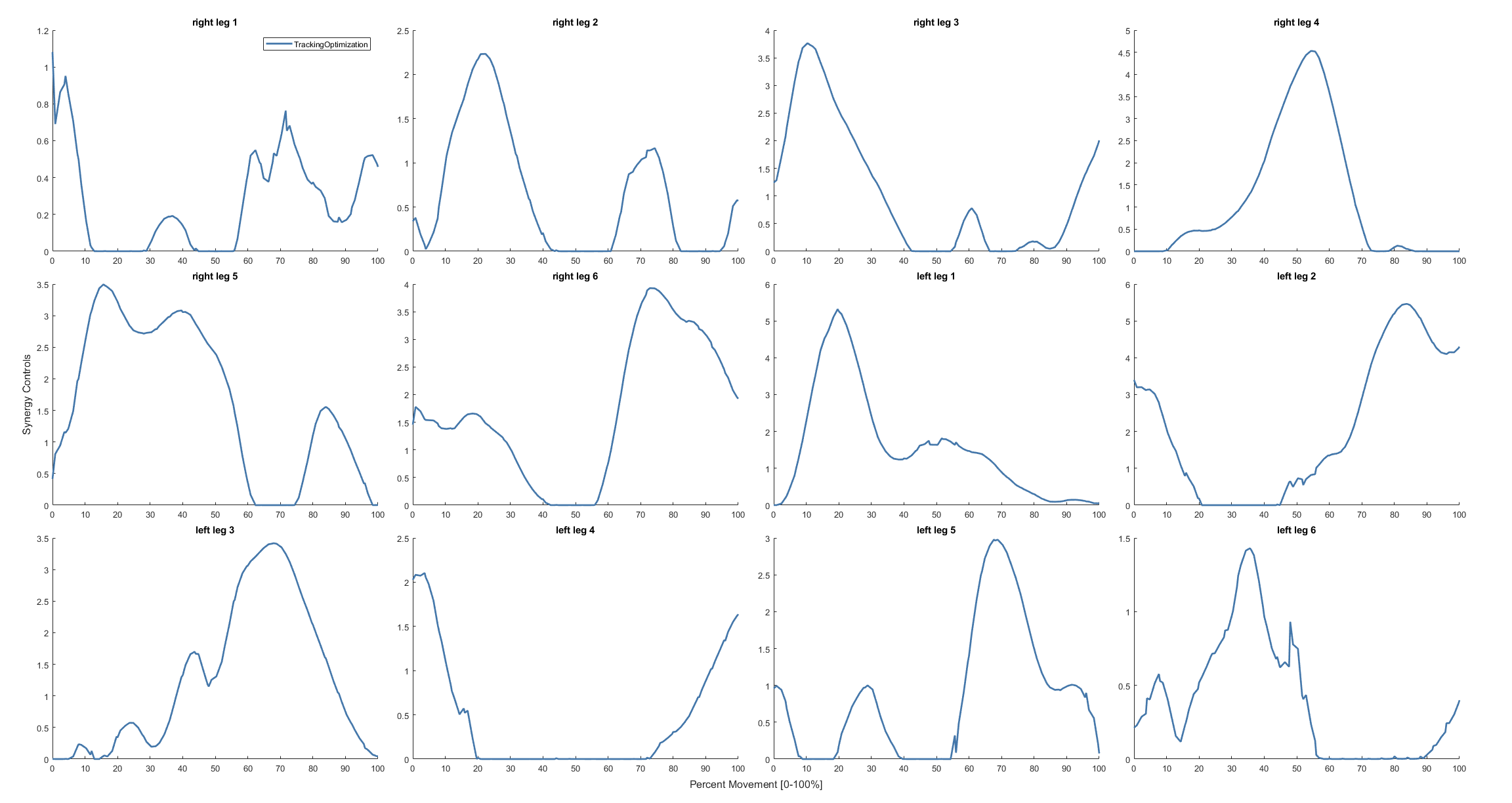

Supplement: Supplement 1 [file media-1.zip › SupplementaryMaterial/TO/synergyControls.png]
